# Highlighting multicomponent reactions as an efficient and facile alternative route in the chemical synthesis of organic-based molecules: a tremendous growth in the past 5 years

**DOI:** 10.3389/fchem.2024.1469677

**Published:** 2024-09-18

**Authors:** Reagan Lehlogonolo Mohlala, Thompho Jason Rashamuse, Elena Mabel Coyanis

**Affiliations:** Advanced Materials Division, Mintek, Randburg, South Africa

**Keywords:** multicomponent reactions, medicinal chemistry, green chemistry, polymerization, synthesis

## Abstract

Since Strecker’s discovery of multicomponent reactions (MCRs) in 1850, the strategy of applying an MCR approach has been in use for over a century. Due to their ability to quickly develop molecular diversity and structural complexity of interest, MCRs are considered an efficient approach in organic synthesis. Although MCRs such as the Ugi, Passerini, Biginelli, and Hantzsch reactions are widely studied, this review emphasizes the significance of selective MCRs to elegantly produce organic compounds of potential use in medicinal chemistry and industrial and material science applications, as well as the use of the MCR approach to sustainable methods. During synthesis, MCRs provide advantages such as atom economy, recyclable catalysts, moderate conditions, preventing waste, and avoiding solvent use. MCRs also reduce the number of sequential multiple reactions to one step.

## 1 Introduction

**FIGURE 1:**
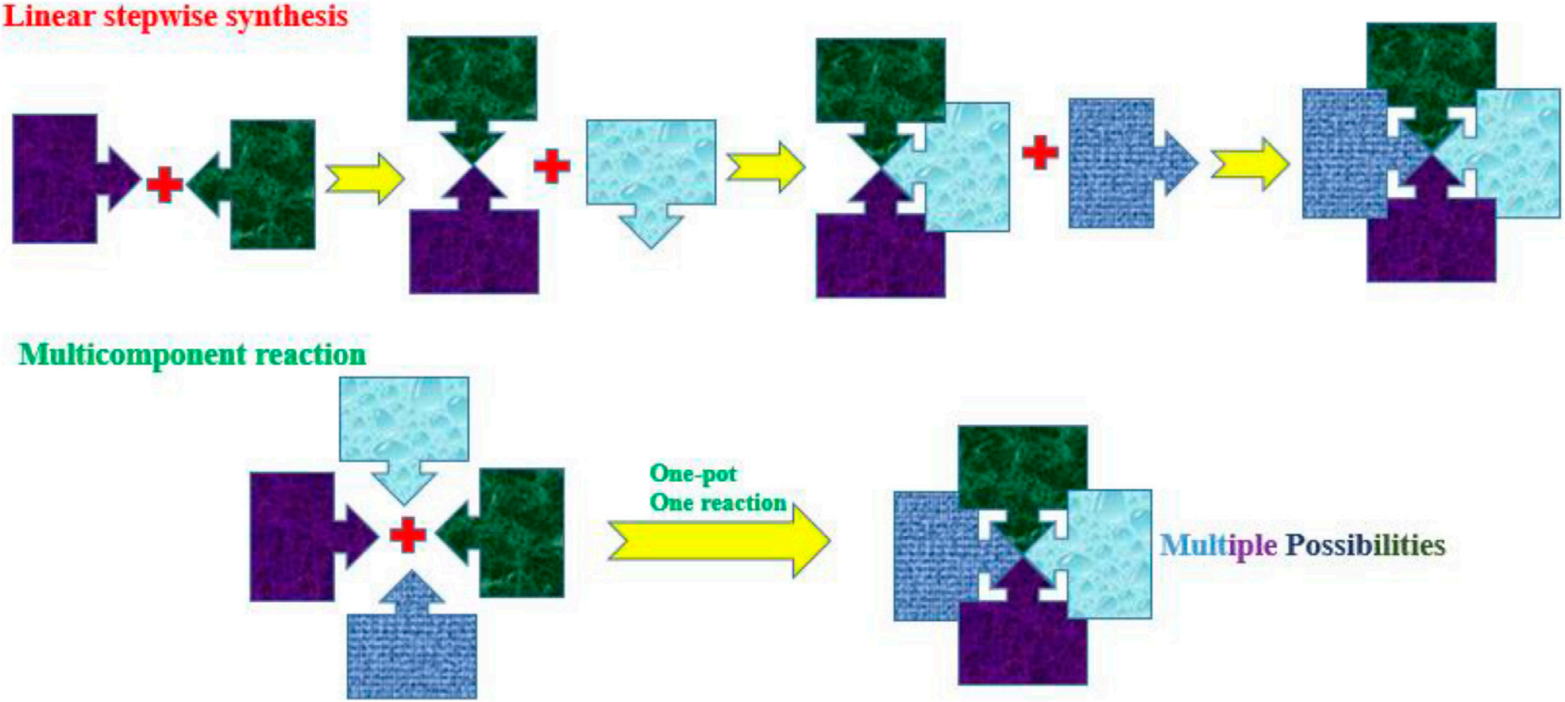
Linear stepwise synthesis vs*.* multicomponent reactions.

Multicomponent reactions (MCRs), also known as one-pot reactions, involve at least three or four reactants of interest to produce the desired single product that contains the building blocks of atoms from each of the reactants (Rocha et al., 2020; Mohlala and Coyanis, 2023; Zhong, 2022; Yazdani et al., 2022). Figure 1 represents the overall practical idea of linear synthesis compared to MCRs, in this case, depicting four separate reactants (building blocks) coming together to generate a single product. MCRs offer efficiency by producing complex molecules and eliminating the need for many purification stages and overall reaction time. The MCR approach offers diversity, which is very helpful in drug discovery and the development of desired molecules by enabling the production of a large range of products from a common set of starting components. MCRs offer sustainability and align with green chemistry principles because of the objectives of minimizing waste generation and the number of stages involved. MCRs are viewed as a practical strategy in synthetic chemistry and offer several advantages over conventional one- or two-component reactions by minimizing the number of sequential multiple reactions necessary and frequently resulting in better yields (Mohlala et al., 2021a; Tang et al., 2018a; Dömling and Ugi, 2000; Nanda et al., 2022).

The use of MCRs for the preparation and synthesis of desired heterocyclic frameworks in medicinal chemistry and drug development is tremendously growing (Wang and Domling, 2021; Wu et al., 2018). This approach offers convenient and accessible methods and procedures. MCRs possess the additional potential to greatly contribute to the development of ecofriendly methods and procedures for pharmaceutical production as they enable the production of complex compounds in a single step (Paprocki et al., 2018; Marset and Guillena, 2022; Mohlala and Coyanis, 2024). MCRs also play a significant role in minimizing the large quantity of waste generated on a daily basis in both pharmaceutical and industrial manufacturing when compared to step-by-step sequential reactions (Yang and Wu, 2018; Shahedi et al., 2021; Paprocki et al., 2018; Tang et al., 2018a).

One of the recognized methods for enhancing “greenness” is the use of MCRs for the production of desired products in the pharmaceutical and industrial fields. The goal of green chemistry is to create chemical processes and products that use fewer or no hazardous ingredients overall. The ground-breaking concept of alternative synthetic reaction schemes to reduce pollution and environmental risks that result from the conventional synthesis technique is conceptualized as green chemistry or sustainable chemistry. The principles of green chemistry include preventing waste, increasing energy efficiency, and using renewable feedstocks. Green, sustainable chemistry practices ensure a safe environment in laboratories, and the use of efficient economical chemical processes is highly recommended for the synthesis and preparation of desired products. MCRs offer a feasible opportunity for the implementation of safe procedures and environmentally friendly manufacturing methods. Green chemistry seeks to minimize the environmental and health impacts of chemical production. MCRs present an excellent opportunity for the realization of a safe environment and friendly practices through greener production processes. The best-known recommended practices and conditions for a green production process include the use of green solvents, operating at mild temperatures, applying biocatalysts, and the practice of catalyst-free reactions.

Multicomponent polymerizations (MCPs) have evolved from MCRs, and these processes produce polymers with well-defined structures, ordered sequences of monomer units, and several functions. One of the benefits of MCPs includes the utilization of elemental sulfur for generating functional materials. The disposal of sulfur is costly, and because it is flammable, its storage is too challenging. Elemental sulfur causes environmental problems such as acid rain (Ota and Richmond, 2011). MCPs provide excellent prospects for the direct use of sulfur due to their intriguing characteristics, which include high efficiency, easy operation, high atom economy, vast structural variety, and gentle conditions (Hu et al., 2016; Wei et al., 2017; Deng et al., 2012; Lee et al., 2013).

MCRs are also applied to overcome the challenges in complicated chiral compound synthesis and are known as asymmetric multicomponent reactions (AMCRs) (Jia et al., 2014). The AMRC is a powerful tool used to achieve diversified, complicated chiral compounds with greater efficiency and atom economy (Xu and Wang, 2014). This approach and strategy proceed by trapping an active intermediate produced from the two reactants with the third component.

In addition to the MCR strategic approach, synthetic chemists have worked on creating peptide analogs in order to circumvent native peptide intrinsic susceptibility to proteolytic degradation and provide novel characteristics and bio-tools for examining protein functions. Currently, naturally occurring peptides have a special place in the pharmaceutical industry for the development of bioinspired compounds (Groß et al., 2016). Within the peptidomimetic framework, β-amino acids have shown exceptional efficacy as precursors for the synthesis of β-peptides and hybrid α/β-polyamino acids, which exhibit enhanced stability toward peptidases and showcase distinct secondary structures and pharmacological properties (Lelais and Seebach, 2004).

MCRs continue to make wonders in synthetic chemistry since they are featured in interesting topics such as solid-phase synthesis and C-H functionalization synthesis. The review positively highlights the contribution of the MCR approach by portraying selected MCRs that efficiently produce organic compounds of potential use in medicinal chemistry and applications in material science, focusing on easier, safer, quicker, more accessible, and cheaper ways to achieve new materials *via* sustainable methods. This review portrays organic synthesis, applications, and the elegant chemistry of MCRs in medicinal chemistry, sustainable chemistry, polymerization, asymmetric catalysis, C-H functionalization, and solid-phase peptide synthesis in the past 5 years (2018–2023).

## 2 Multicomponent reactions in medicinal chemistry, green chemistry, polymerization, asymmetric catalysis, C-H functionalization, and peptide synthesis

### 2.1 Medicinal chemistry

**SCHEME 1:**
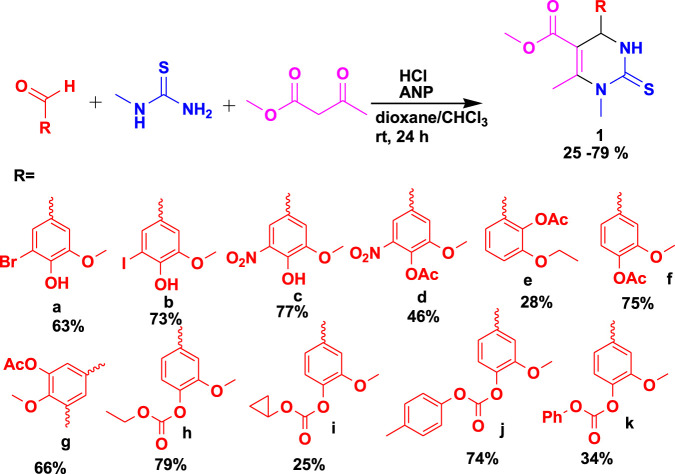
Synthesis of bioactive THPM derivatives.

Medicinal chemistry, biology, and pharmacology play important roles in the intricate process of discovering new medicines. Nevertheless, this process inescapably depends on a crucial organic synthesis base to guarantee the availability of the necessary molecules. From hit discovery to production, this synthetic activity permeates all aspects of drug discovery/medicinal chemistry and has recently made tremendous strides (Blakemore et al., 2018; Gioiello et al., 2020). A different approach to target synthesis is offered by MCRs, which are characterized by their “one-pot” reaction, the promotion of a very broad chemical space based on readily available basic building blocks, and the ability to tackle difficult sustainability-related issues. Consequently, the MCR approach has the potential to be highly helpful in the identification and synthesis of biologically active “drug-like” compounds. In a way, the interest is to cover specific instances of how the MCR approach has been used in medicinal chemistry, including drug discovery, drug synthesis, screening libraries, and biopharmaceutical applications. Buskes et al. (2023) reported the synthesis of complex chemotypes using the MCR approach. The biologically active tetrahydropyrimidine (THPM) (**1**) derivatives were discovered after the three-component reaction (3CR) of vanillic aldehydes with *N*-methylthiourea and methyl acetoacetate using 2-amino-1-(4-nitrophenyl)-1,3-propanediol (ANP) and hydrochloric acid, as shown in Scheme 1 (Milovic et al., 2022). It has been reported that these bioactive THPMs exhibit good biological activities such as antimicrobial, anticancer, and glucosidase inhibitory effects. These compounds were further evaluated against five strains of bacteria and fungi, and the results were found to be effective. The compounds were achieved in 24 h at room temperature, with the highest yield reaching 79%.

**SCHEME 2:**
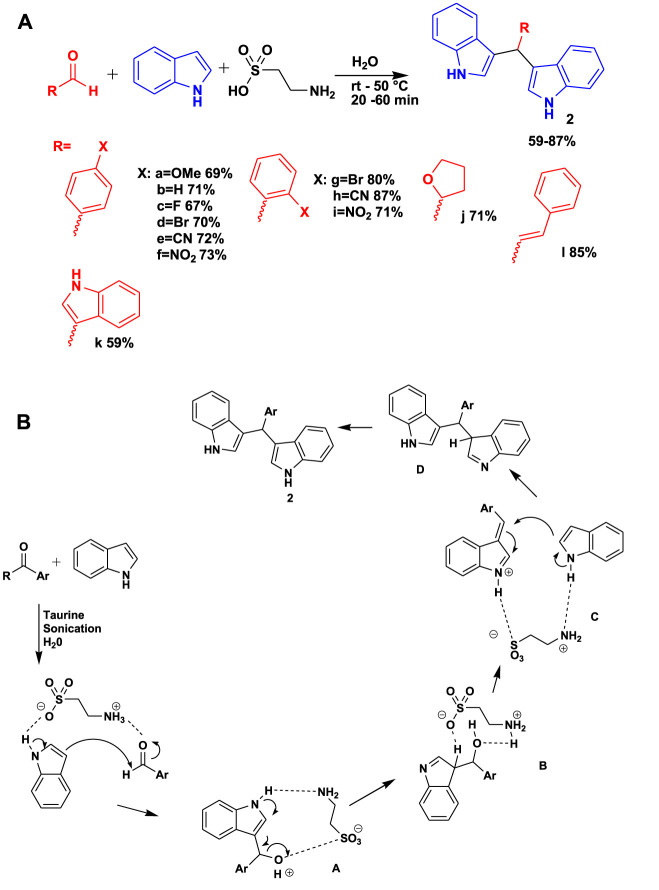
**(A)** Synthesis of 3,3-bis(indolyl)methanes (BIMs). **(B)** Mechanism for the synthesis of 3,3-BIMs.

The 3CR of *p*-methoxybenzaldehyde and indole in the presence of taurine using water as a solvent under sonification yielded potential bioactive 3,3-bis(indolyl)methanes (BIMs) (**2**) and their derivatives, as shown in Scheme 2 (Chavan et al., 2022). This set of compounds has the potential to bind to antineoplastic drug targets and spindle motor protein kinesin Eg5. The benefit of using this MCR includes a short reaction time, mild room temperature conditions, and the use of water as a green solvent. Scheme 2B shows a possible reaction mechanism of water-mediated, taurine-catalyzed indole and *p*-methoxybenzaldehyde for the synthesis of 3,3-bis(indolyl)methanes. The reaction proceeds by a taurine-activated aldehyde moiety inviting the activated indole through an addition reaction, leading to adduct A. Taurine caused intermediate A to undergo protonation and then dehydration, resulting in the formation of another intermediate, benzylidene-3H-indole C, via intermediate B. The subsequent activation of indole led to the formation of bisindole moiety D, which subsequently experienced imine–enamine tautomerization to yield the desired compound 2, as shown in Scheme 2B.

**SCHEME 3:**
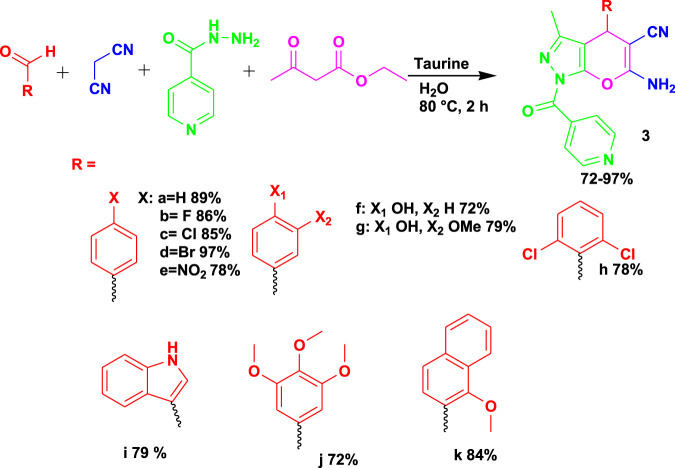
Synthesis of therapeutic dihydropyrano[2,3-c]pyrazoles.

Mali et al. (2021) reported the four-component reaction (4CR) of benzaldehyde with malononitrile in the presence of hydrazine hydrate and ethyl acetoacetate using taurine as the catalyst with solvents such as water, ethanol, acetonitrile, and toluene for the synthesis of therapeutic dihydropyrano[2,3-*c*]pyrazoles (**3**), as shown in Scheme 3. The *in silico* analysis of dihydropyrano[2,3-*c*]pyrazoles demonstrated their potential to bind to variants of dihydrofolate reductase in pathogenic *Staphylococcus aureus* strains. In addition to the good biological activities, the reaction is efficient and facile, occurs under mild conditions, and uses water as a solvent, with a reaction time of 2 h.

**SCHEME 4:**
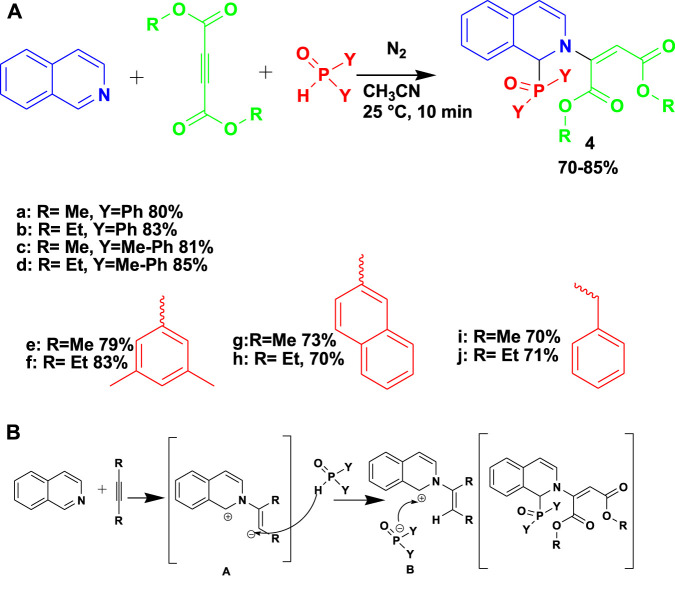
**(A)** Synthesis of aryl phosphinoyl-functionalized dihydroisoquinoline derivatives. **(B)** Mechanism for the synthesis of aryl phosphinoyl-functionalized dihydroisoquinoline derivatives

The biologically active arylphosphinoyl-functionalized dihydroisoquinoline (**4**) derivatives were synthesized by the 3CR of isoquinoline with dialkyl acetylenedicarboxylates and phosphine oxides or ethyl phenyl-*H*-phosphinate under a nitrogen atmosphere using acetonitrile as a solvent, as shown in Scheme 4 (Popovics-Toth et al., 2022). This set of compounds showed good activity against human promyelocytic leukemia (HL-60) cells and Gram-positive *Bacillus subtilis* strains. Unlike those at high temperatures and under hazardous conditions, the conversion of this reaction was done at room temperature, and the product was achieved in an hour.

The proposed mechanism for the formation of phosphorylated dihydroisoquinoline proceeds by a Reissert-type reaction.

To generate a zwitterion intermediate (A), isoquinoline is added as a nucleophile to activated acetylene in the first step. Next, the secondary phosphine oxide protonates the carbanionic center of intermediate A. Lastly, the nucleophilic addition of the phosphorus reagent results in the formation of product 4.

**SCHEME 5:**
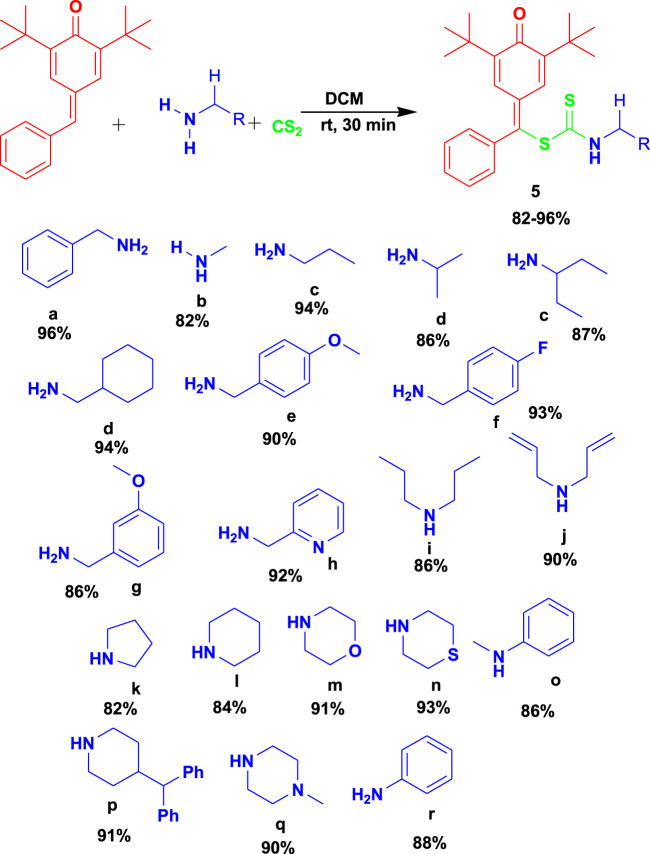
Synthesis of biologically active S-benzyl dithiocarbamates.

Venkatesh et al. (2022) reported the 3CR of *para*-quinone methides with amines and carbon disulfide using dichloromethane (DCM) as a solvent from 0°C to room temperature for the synthesis of biologically active *S*-benzyl dithiocarbamate (**5**), as shown in Scheme 5. The biologically active compounds were attained in excellent yields and were reported to have some acetylcholinesterase (AChE) inhibitory and antioxidant activities. This set of reactions occurs under safe conditions (room temperature and catalyst-free) and in a short time with excellent yields.

**SCHEME 6:**
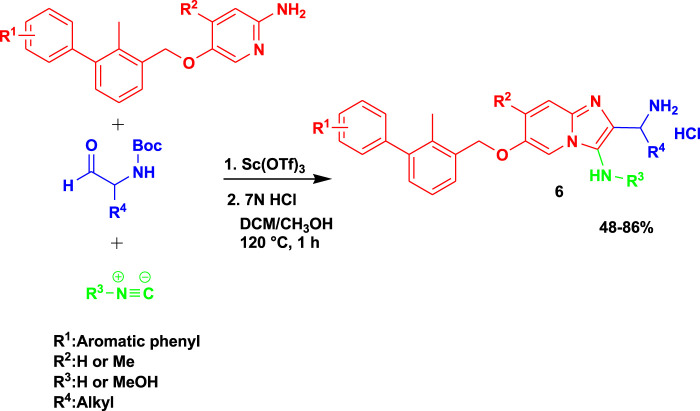
Synthesis of imidazo[1,2-a]pyridine-based inhibitors.

Groebke–Blackburn–Bienaymé 3CR (GBB-3CR) of the aminopyridine component with aldehyde and isocyanide in dichloromethane and methanol as solvents under microwaving at 120°C in the presence of scandium triflate yielded imidazo[1,2-*a*]pyridine-based (**6**) inhibitors, as shown in Scheme 6 (Butera et al., 2021). The synthesized imidazopyridine inhibitors showed potential as candidates with low micromolar PD-L1 affinities, and the result paves the way for biologically active scaffolds, leading to a class of PD-L1 antagonists. Despite the good biological activities, this set of reactions under microwave assistance yielded the final products of 11 good-to-excellent yields (48%–86%).

**SCHEME 7:**
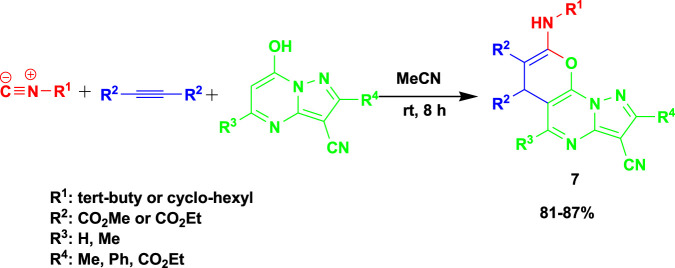
Synthesis of pyrano[3,2-e]pyrazolo[1,5-a ]pyrimidines.

Vahedi et al. (2023) reported the 3CR of alkyl isocyanides with dialkyl acetylenedicarboxylates and 7-hydroxy pyrazolo[1,5-*a*]pyrimidines at room temperature using acetonitrile as a solvent to obtain biologically active pyrano[3,2-*e*]pyrazaolo[1,5- *a*]pyrimidine (**7**) in good yields, as shown in Scheme 7. One of the reported synthesized compounds exhibited excellent potential as an anticancer agent with an IC_50_ value of 19.70 ± 0.89 μM, comparable to the standard drug etoposide (IC_50_ = 18.71 ± 1.09 μM). Additionally, the derivatives containing a phenyl group demonstrated promising antioxidant characteristics by having a high free radical scavenging effect (IC_50_ = 12.12 ± 0.40 μM), which was equivalent to the ascorbic acid (IC_50_ = 11.85 0.30 μM) benchmark for antioxidants. This set of reactions uses the recommended solvent acetonitrile at room temperature to produce 16 examples of pyrano[3,2-*e*]pyrazaolo[1,5-*a*]pyrimidine.

The probable mechanism of the reaction is shown in Scheme 2. Vinylisonitrilium cation 7 is first generated by Michael’s addition of alkyl isocyanide 1 to acetylenic ester 2 and then protonating intermediate 5 by pyrazolo[1,5-a]pyrimidine 3 as an OH acid. This positively charged intermediate may be attacked by the pyrazolo[1,5-a]pyrimidine anion 6 to produce ketene-imine 8, which can then be tautomerized to intermediate 9. Cyclization of intermediates 9 to 10 (two resonance forms 10I and 10II) followed by a 1,3-proton exchange yielded desired product 4.

**SCHEME 8:**
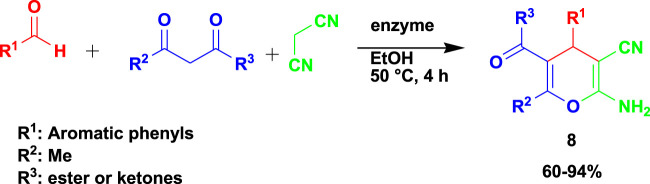
Synthesis of biologically active 2-amino-4H-pyrans.

Yang et al. (2020) reported the synthesis of biologically active 2-amino-4*H*-pyrans (**7**) in a 3CR of aldehyde with malononitrile and ethyl acetoacetate using lipases as catalysts and different solvent media, as shown in Scheme 8. The synthesized compounds were subjected to *in vitro* antitumor activities against three cell lines (A549, HeLa, and HepG-2) and displayed good activity against tumor cells. One of the compounds was reported to have an IC_50_ value of 0.0517 μM in A549 cells as an antitumor agent. Ethanol, as a green solvent with a green catalyst enzyme and mild condition of only 4 h to produce desired products, is highly recommended from the MCR that resulted in good-to-excellent yields.

**SCHEME 9:**
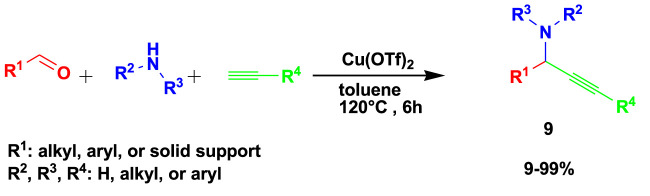
Synthesis of anticancer propargylamines.

The anticancer propargylamines (**9**) were prepared by the 3CR of an aldehyde, amine, and alkyne using toluene as a solvent in the presence of Cu(OTf)_2_ as a catalyst, as shown in Scheme 9 (Martinez-Amezaga et al., 2020). Toluene is one of the recommended solvents because it is less hazardous than benzene. The yields range from low to excellent percentages produced in a maximum time of 6 h.

This set of compounds showed a high degree of selectivity, with an index higher than 3 for triple-negative breast cancer cells and a very interesting selectivity of 41.17 for pancreatic cancer cells. About 12 compounds exhibited an inhibition of viability greater than 50% and were considered to have an antitumor effect on 4T1 and PANC-1 cells. The toxicity effect on the MC3T3el normal cell line was tested for evaluation against the tumor cells.

**SCHEME 10:**
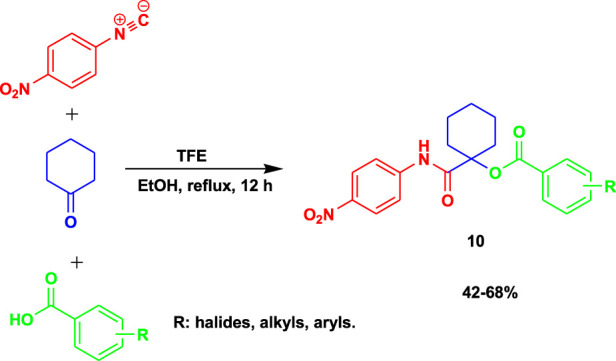
Synthesis of α-acyloxycarboxamides.

The caspase-dependent apoptotic inducers α-acyloxycarboxamides (**10**) were prepared from *p*-nitrophenyl isonitrile, cyclohexanone, and various carboxylic acids using 2,2,2-trifluoroethanol (TFE) as a catalyst under reflux in ethanol, as shown in Scheme 10 (Ayoup et al., 2019). The IC_50_ test results of this set of compounds of α-acyloxycarboxamides showed potential and safety (0.185 ± 0.01 at 100 µM) compared to doxorubicin (0.0266 ± 0.005 at 100 µM) based on IC_50_ values during cytotoxicity against normal human fibroblasts and anticancer activities against MCF-7 breast, NFS-60 myeloid leukemia, and HepG-2 liver utilizing MTT assay.

The 3-MCR occurs in an ethanol solution at mild temperatures to produce average yields of *α*-acyloxycarboxamides.

**SCHEME 11:**
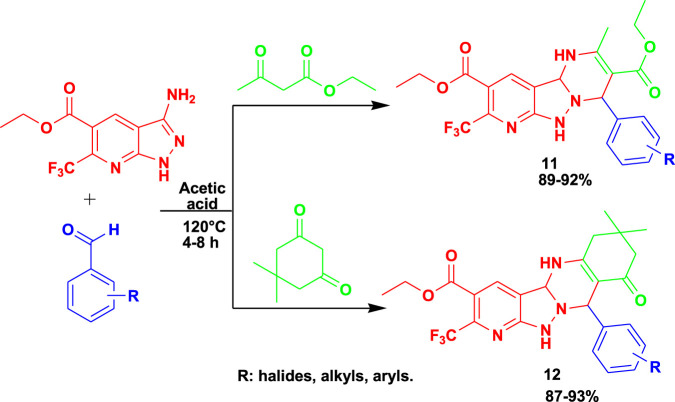
Synthesis of pyrimidine-fused pyrazolo [3,4-b] pyridine derivatives (11) and hexahydroquinazoline-fused pyrazole[3,4-b]pyridine derivatives (12).

The 3CR of 3-amino-5-carbethoxy-6-trifluoromethyl pyrazolo[3,4-*b*] pyridine with aldehyde and ethyl acetoacetate /dimedone using acetic acid yielded biologically active pyrimidine-fused pyrazolo[3,4-*b*] pyridine (**11**) derivatives and hexahydroquinazoline-fused pyrazole [3,4-*b*] pyridine (**12**) derivatives, as shown in Scheme 11 (Pradeep et al., 2019). The yields of the following MCRs were found to be excellent under mild conditions after 8 h. Compounds **11** and **12** exhibited very good antibacterial, antifungal, and antibiofilm activity against Gram-positive and Gram-negative bacterial strains. Compounds **11** and **12** exhibited promising activity against *Klebsiella planticola* MTCC 530 at a minimum inhibitory concentration (MIC) value of 3.9 μg/mL. In addition, some of these derivatives showed good activity against *S. aureus* MLS-16 MTCC 2940 at a MIC value of 3.9 μg/mL.

**SCHEME 12:**
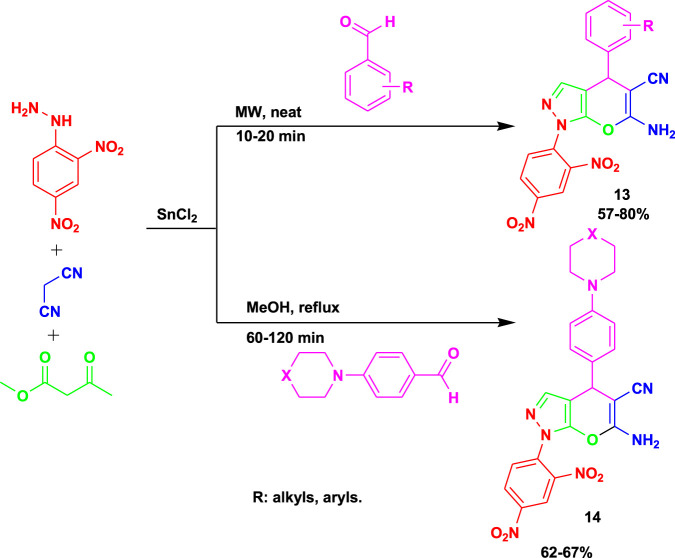
Synthesis of biologically active 6-amino-1-(2,4-dinitrophenyl)-4-phenyl-1,4-dihydropyrano[2,3-c]pyrazole-5-carbonitrile derivatives.

Vasava et al. (2019) synthesized biologically active 6-amino-1-(2,4-dinitrophenyl)-4-phenyl-1,4-dihydropyrano[2,3-c]pyrazole-5-carbonitrile (**13**) derivatives via the 4CR of 2,4-dinitrophenyl hydrazine, malononitrile, ethyl acetoacetate, and aromatic aldehyde derivatives using SnCl_2_ as a catalyst, as shown in Scheme 12. The derivatives of compound **12** were obtained via microwaving between 10 and 20 min, whereas compound **14** was achieved under methanol reflux between 1 and 2 h. The reaction conditions were mild, involving a neat reaction and requiring less time to achieve a good-to-excellent yield from the MCR.

The compounds performed well *in vitro* biological evaluations as antibacterial and antituberculosis agents. The derivatives of compounds **13** and **14** were screened against multi-drug resistance (MDR-TB) and resistance to first-line anti-TB drugs in clinical strains, and only one derivative of compound **14** showed moderate activity, with a MIC value of 50 mg/mL. The derivative of compound **13** exhibited outstanding antibacterial activity against *E. coli* and *P. aeruginosa* at a MIC value of 12.5 mg/mL.

**SCHEME 13:**
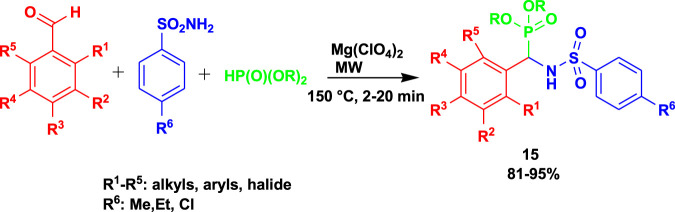
Synthesis of biologically active α-sulfonamidophosphonate derivatives.

Bhagat et al. (2019) reported the α-sulfonamido phosphonates (**15**) as anti-*Mycobacterium tuberculosis* H37Rv agents. The α-sulfonamidophosphonates (**15**) were obtained after treating 4-methoxybenzaldehyde with 4-methylbenzenesulfonamide and dimethyl phosphite using magnesium perchlorate under microwave irradiation, as shown in Scheme 13. This class of compounds exhibited good MIC values of 1.56 μg/mL and 3.125 μg/mL. In addition, some of the active compounds are non-toxic to RAW 264.7 (mouse leukemic monocyte macrophage) cell lines. The MCR methods gave rise to excellent yields within a short time, 20 min, using safe catalysts.

**SCHEME 14:**
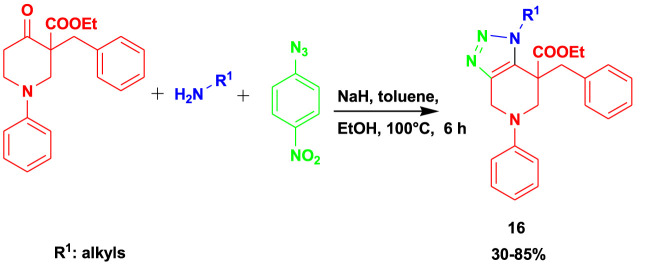
3CR for the synthesis of biologically active 1,2,3-triazole.

Karypidou et al. (2018) reported the synthesis of biologically active 1,2,3-triazole (**16**) from the 3CR of oxopiperidine carboxylate with primary amines and 4-nitrophenyl azide using toluene as a solvent at 100°C, as shown in Scheme 14. This set of compounds demonstrated promising antiviral activity against human coronavirus 229E with EC_50_ = 8.95 µM. Under mild conditions, the MCRs gave average-to-good yields using sodium hydride as a base and toluene as a solvent.

**SCHEME 15:**
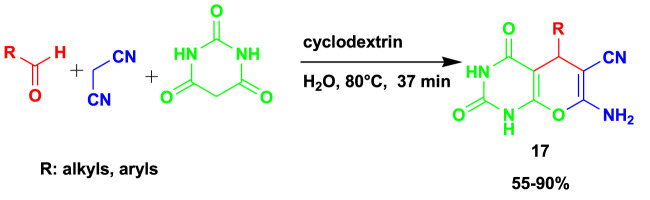
3CR for the synthesis of pyrano[2,3-d]-pyrimidinone derivatives.

The 3CR of benzaldehyde with malononitrile and barbituric acid gave rise to biologically active pyrano[2,3-*d*]-pyrimidinone (**17**) derivatives using *β*-cyclodextrin as a catalyst and water as a solvent, as shown in Scheme 15 (Chate et al., 2018). The *in vitro* antimicrobial activity evaluations of pyrano[2,3-*d*]-pyrimidinone (MIC 50 ± 0.96 lg/mL, against *B. subtilis*) exhibited good results compared to ciprofloxacin, used as the reference drug. This method gave up to 90% yield under mild conditions, using water as the green solvent.

### 2.2 Green chemistry

**FIGURE 2:**
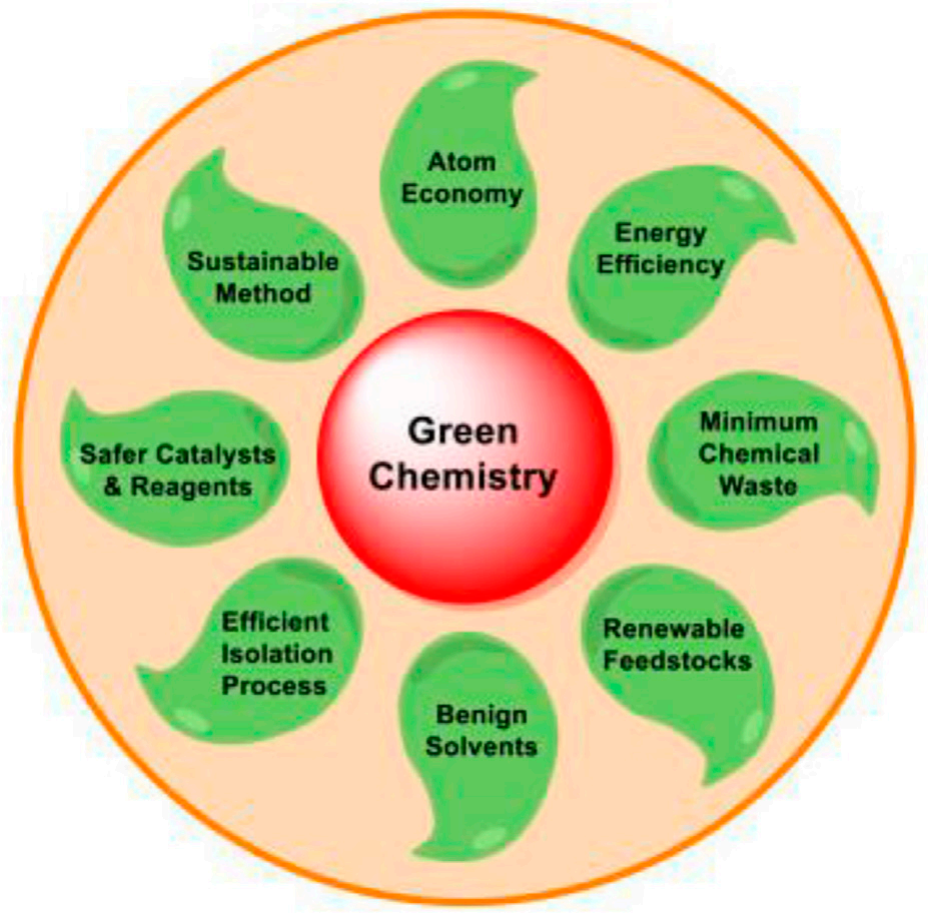
Green chemistry principles.

Green chemistry pertains to the advancement of chemical products and processes that reduce or eliminate the utilization or creation of dangerous substances. Green chemistry encompasses the entirety of the life cycle of a chemical product, which includes its creation, utilization, and ultimate disposal. Green, sustainable chemistry practices provide a safe environment in laboratories. The use of efficient and economical chemical processes is highly recommended for the synthesis and preparation of desired products. One of the recognizable methods for enhancing “greenness” is the use of MCRs in the production of medicinal and industrial products. MCRs offer a great chance to implement safe practices and environmentally safe methods. The use of green solvents, solvent-free reactions, working at modestly safe temperatures, utilizing biocatalysts, photocatalysis, safe catalysts, and catalyst-free reactions are some of the most well-known suggested practices and conditions for a green production process. The significant benefit of green chemistry is undeniable, according to regulatory bodies, federal and state governments, and scientific societies. The 12 Principles of Green Chemistry, first outlined by Anastas and Warner (1998) in 1998 and later revised by Asfaw et al. (2011) and Anastas and Eghbali (2010), serve as the norm for green chemistry practice and sustainable production (Figure 2).

**SCHEME 16:**
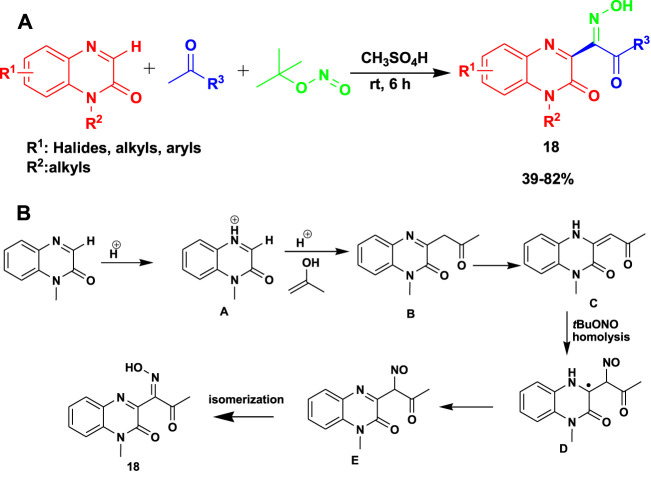
**(A)** Greener chemical synthesis of (E)-quinoxalinone oximes. **(B)** Mechanism for the greener chemical synthesis of (E)-quinoxalinone oximes.

The 3CR of quinoxalinones with methyl ketones and *tert*-butyl nitrite in the presence of methanesulfonic acid at ambient temperature gave rise to (*E*)-quinoxalinone oximes (**18**) (Xu et al., 2021). The reactions occurred under greener chemical production with transition metal-free conditions to yield moderate-to-good products, as shown in Scheme 16. This reaction gave up to 82% yield at room temperature after 6 h.

Quinoxalinones are protonated to produce the first iminium ion (A). Meanwhile, in an acidic environment, acetone changes into the enol form. This is followed by the reaction of A and enol to obtain B. Therefore, compound B is converted to C through an oxidative dehydrogenation process. The homolysis of TBN yields tert-butoxy (tBuO) and nitroso (·NO) radicals. The next step involves the attack from the nitroso (NO) radical to the C=C bond of C to obtain intermediate D, which is then oxidized to intermediate E by tert-butoxy (tBuO). This is followed by the final step, which involves isomerization to obtain the product.

**SCHEME 17:**
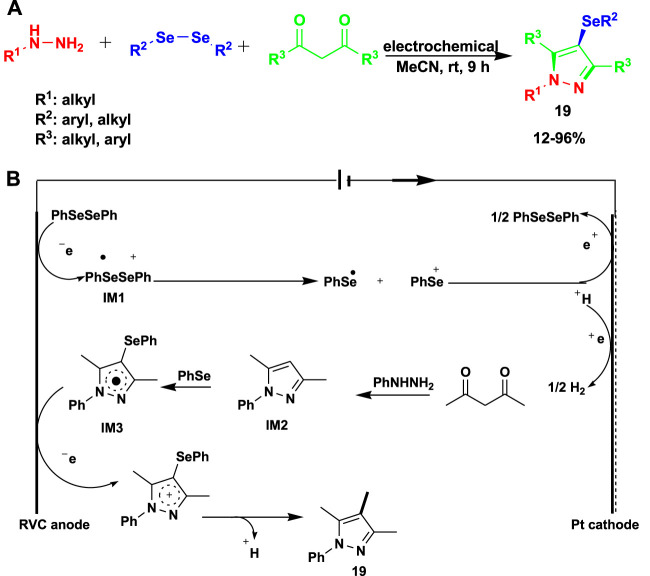
**(A)** Electrochemical multicomponent reaction for the synthesis of 4-selanylpyrazoles. **(B)** Mechanism for the synthesis of 4-selanylpyrazoles using the electrochemical multicomponent reaction.

Wu et al. (2021) reported the electrochemical multicomponent reaction of phenylhydrazine with diphenyl diselenide and 2,4-pentanedione using a reticulated vitreous carbon (RVC) anode/Pt plate cathode system in acetonitrile as a solvent to obtain 4-selanylpyrazoles (**19**). This method requires no catalyst or oxidant and proceeds under mild conditions to obtain good-to-excellent yields, as shown in Scheme 17.

A possible mechanism for the electrochemical multicomponent reaction is shown in Scheme 17B. In order to produce an intermediate, free radical cation (IM1) diphenyl diselenide 3a is first oxidized on the anode surface. The unstable IM1 then breaks down into a cation and a radical of phenyl selenium. Then, pyrazole, which is produced *in situ* by the cyclo-condensation of 1,3-diketone and hydrazine, reacts with the phenyl selenium radical to produce pyrazole radical IM2, which is then further oxidized at the anode to produce pyrazole cation intermediate IM3. Eventually, the deprotonation of intermediate IM4 and the subsequent proton release result in the generation of the final 4-selanylpyrazole product (19).

**SCHEME 18:**
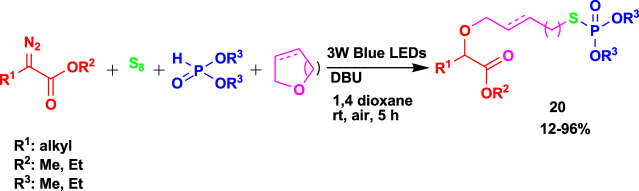
4CR for the synthesis of S-alkyl phosphorothioate.

The 4CR of α-diazoesters, elemental sulfur, *H*-phosphonates, and cyclic ethers yielded *S*-alkyl phosphorothioates (**20**), as shown in Scheme 18 (Qu et al., 2022). The reaction to achieve the products was done through visible light using 1,8-diazabicyclo(5.4.0)undec-7-ene (DBU) as an additive under mild conditions.

**SCHEME 19:**
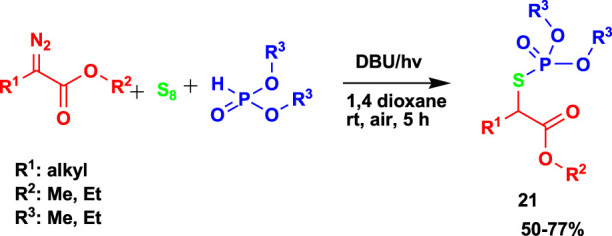
3CR for the synthesis of S-alkyl phosphorothioate.

Furthermore, Qu et al. (2022) studied the 3CR of α-diazoesters, elemental sulfur, and *H*-phosphonates to generate *S*-alkyl phosphorothioate (**21**) in moderate-to-good yields using visible light and base DBU, as shown in Scheme 19. Despite using other solvents, recommended green solvents such as dimethyl sulfoxide, tetrahydrofuran, and acetonitrile were used.

**SCHEME 20:**
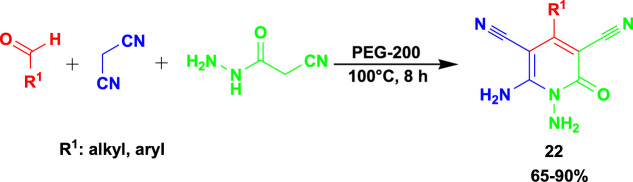
Synthesis of functionalised N-amino-3-cyano-2-pyridone.

The 3CR of aldehydes with cyanoacetohydrazide and malononitrile or ethyl cyanoacetate yielded functionalized *N*-amino-3-cyano-2-pyridone (**22**) using a biodegradable green solvent PEG-200 and other solvents, such as water, ethanol, and glycerin, and neat conditions with temperatures ranging from room temperature to 100°C (Scheme 20) (Das et al., 2023). PEG-200 is one of the good solvents considered for sustainable chemistry synthesis since it is non-toxic, economical, easily available, and robust to promote this cascade method.

**SCHEME 21:**
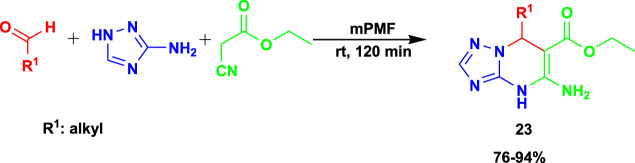
3CR for the synthesis of dihydro-[1,2,4]triazolo [1,5-a]pyrimidines.

The 3CR of 3-amino-1,2,4-triazole, aldehyde, and ethyl cyanoacetate yielded dihydro-[1,2,4]triazolo[1,5-*a*]pyrimidines (**23**) under greener chemical production using poly-melamine formaldehyde (mPMF) as the catalyst (Scheme 21) (Khaligh and Mihankhah, 2022). The reactions were performed at room temperature under solvent-free conditions. The catalyst was reported to be recyclable for at least five runs while still providing good efficiency.

**SCHEME 22:**
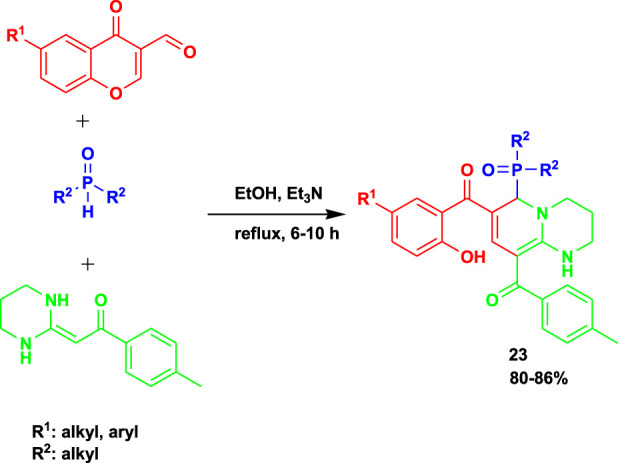
Synthesis of highly functionalized 2-(diarylphosphoryl)-1,2-dihydropyridine derivatives.

Li et al. (2022) reported the 3CR of heterocyclic ketene aminals, phosphine oxides, and 3-formylchromones to obtain highly functionalized 2-(diarylphosphoryl)-1,2-dihydropyridine (**24**) derivatives, as shown in Scheme 22. The investigation revealed that when the reactions were performed using propylene carbonate (PC) as a solvent and trimethylamine as a promoter, the highest yields were achieved.

**SCHEME 23:**
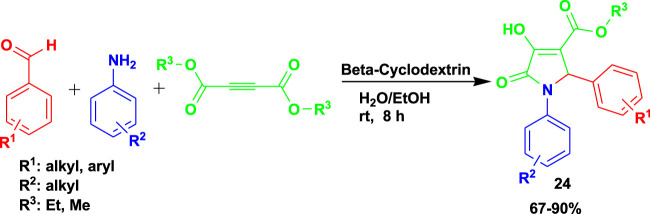
Synthesis of pyrrolidine-2-one using β-cyclodextrin.

Paul et al. (2023) developed a sustainable and ecofriendly method for the synthesis of pyrrolidine-2-one (**24**) using a water-soluble supramolecular β-cyclodextrin as a catalyst. The 3CR of aldehydes with amines and dimethylacetylenedicarboxylate (DMAD) was done at room temperature using a water–ethanol medium to obtain the products with good-to-excellent yields, as shown in Scheme 23.

Kerru et al. (2021) developed an efficient and sustainable method after setting a 4CR of 1*H*-1,2,4-triazole-3-amine with aldehydes, methylene compounds, and diethyl acetylenedicarboxylate using water as the solvent under microwave irradiation conditions to obtain 1,2,4-triazole-tagged 1,4-dihydropyridine (**25**) derivatives (Supplementary Scheme S1). The desired products were achieved with excellent yields ranging from 94% to 97% and high selectivity at room temperature. The solvent effect was investigated by testing polar-aprotic solvents (acetonitrile, dichloromethane, and tetrahydrofuran), which resulted in lower yields than polar-protic solvents (acetic acids, water, methanol, and ethanol).

Ma et al. (2019) reported the greener chemical diastereoselective synthesis of tetrahydropyrrolo[1,2-*d*][1,4]benzodiazepines (**26**) and tetrahydropyrrolo[1,2-*d*][1,4]diazepinones (**27**), as shown in Supplementary Scheme S2. The 4CR of L-alanine ethyl ester hydrochloride with *N*-ethylmaleimide and 2-azidobenzaldehyde in the presence of phenacyl bromide or phenylglyoxylic acid using acetonitrile as a solvent under microwave conditions gave good yields.

The condensation of 2-azidobenzaldehyde and L-alanine ethyl ester yielded azomethine ylide (A), followed by [3 + 2] cycloaddition to yield pyrrolidine (B). The N-alkylation of phenacyl bromide occurs, followed by the Staudinger/aza-Wittig reaction *via* phosphanylidene to produce tetrahydro-pyrrolobenzodiazepine derivatives (27).

El-Lateef et al. (2023) reported an efficient and sustainable method by setting an FF4CR of *p-*formylphenyl-4-toluenesulfonate with ethyl cyanoacetate, ammonium acetate, and acetophenone derivatives using ethanol as a solvent under microwave irradiation to yield pyridine (**28**) (Supplementary Scheme S3). This method gave even better yields with good atom economy and pure products in a short reaction time, which is recommended for the synthesis of heterocyclic compounds or organic-related materials.

### 2.3 Polymerizations

The approach of MCR chemistry toward the production of functionalized polymers with good properties for advanced material use is not well exploited. However, recently, the application of MCRs in polymer chemistry is slowly gaining interest. Polymeric materials possess distinguishing and unique thermal, fluorescence sensing, mechanical, chemical, and optoelectronic properties, which enhance their practical chemical applications. MCP has significantly accelerated the growth of polymer science and advanced material sectors. Some of the heteroatom polymers derived from MCP have been applied in various fields, including water treatment (Tian et al., 2018), flame retardants (Velencoso et al., 2018), and fluorescent sensors (Wang et al., 2020). The interest in using MCP for the production of polymers is gradually growing, and it brings some interesting benefits such as multiple sequential stepwise reductions, atom economy, and mild conditions.

The three-component reaction of alkyne, sulfonyl azide, and *N,N*-dialkyloxy formamide dialkyl acetal yielded functional polymers (**29**), as shown in Supplementary Scheme S4 (Su et al., 2021a). The polymerizations leading to functional polymers were accomplished using copper bromide as a catalyst, bis(diphenylphosphino)ethane as a ligand, and molecular sieves (4 Å) under N_2_ with DCM as a solvent at room temperature for 1 h. The facile method of polymerization occurs under mild conditions, which provides high polymerization efficiency and stereoselectivity. The multiple functional polymers produced show good activity in acid–base responsive fluorescence, visualization of food spoilage, efficient gold enrichment and recovery, and lysome-specific cell imaging, and are also highly selective with a sensitive Au^3+^ sensor.

Xu et al. (2019) treated an alkyne, sulfonyl azide, and 2-hydroxybenzonitrile in the presence of copper chloride and trimethylamine under N_2_ using dichloromethane as a solvent to achieve functional polymers (**30**), as shown in Supplementary Scheme S5. The iminocoumarin/quinolone-containing poly(*N*-sulfonylimine)s obtained had molecular weights up to 37,700 g/mol and a 96% yield. The fluorescence poly(*N*-sulfonylimine) (**30**) shows good activities such as sensitivity and detection of Ru^3+^ and some antibacterial properties. Gram-positive bacterium *S. aureus* and Gram-negative bacterium *E. coli* were selected, and the results showed an inhibitory effect on the growth of *S. aureus* and a calculated inhibition ratio of 58% when *S. aureus* was incubated with 32 μM of compound **30** for 12 h.

The reaction of sulfur, diamines, and diisocyanides at room temperature using dimethylformamide and toluene as solvents yielded functional polythioureas (**31–32**) under catalyst-free conditions in air with 100% atom economy, as shown in Supplementary Schemes S6A, B (Tian et al., 2018). The scope of this polymerization was explored to obtain more products of polythioureas that are well characterized. The polythioureas had molecular weights up to 242,500 g/mol with excellent yields of up to 95%. This set of polythioureas displayed distinct activities such as the detection of mercury pollution with high sensitivity and selectivity and the ability to efficiently clean Hg^2+^ to obtain clean drinking water.

Polymers with aggregation-induced emission (AIE) have properties with advantages such as structural diversity, multi-functionalities, efficient solid-state fluorescence, and good processability, as shown in Supplementary Scheme S7 (Su et al., 2021b). The 3CR of diynes (**a**), dialdehydes (**b**), and secondary amines (**c**) at 140°C using indium trichloride as a catalyst and *o*-xylene as a solvent yielded polymers (**33**) with AIE, as shown in Supplementary Scheme S7A. Supplementary Scheme S7B shows the successful 3CR polycoupling of diynes (**a**), aldehydes (**b**), and primary amines (**c**) using copper chloride as a catalyst and toluene as a solvent at 100°C to obtain polymers (**34**). The metal-free 3CR polycoupling of diynes (**a**), aldehydes (**b**), and urea (**c**) to produce polymers (**35**) with AIE was achieved at 90°C using acetonitrile as a solvent, as shown in Supplementary Scheme S7C.

The reaction of isocyanides, amines, and elemental selenium via 3CR to form selenourea polymers (**36**) using dichloromethane as a solvent at room temperature was reported by Tuten et al. (2018), as shown in Supplementary Scheme S8. The reaction proceeds with isocyanide reacting with selenium powder to form isoselenocyanates, and this process has 100% atom economy. Isoselenocyanates are formed *in situ* from elemental selenium and isocyanides, leading to the reaction with amines yielding selenourea polymers.

The polythioamides (**37**) were achieved by Zhang et al. (2022) from room temperature to 40°C under catalyst-free conditions by treating sulfur, aromatic alkynes, and diamines using polar aprotic solvents such as DMSO, DMF, and DMAc, as shown in Supplementary Scheme S9. The well-defined structures of polythioamides were achieved with good-to-high yields and high molecular weights of up to 95,100 g/mol. The functional polythioamides (**37**) exhibit interesting photo-physical and photo-chemical properties.

The reaction of bis(*N*-sulfonyl aziridine)s, tosyl isocyanate, and diols using organocatalyst (1-methyl-2,3,4,6,7,8-hexahydro-1*H*-pyrimido[1,2-*a*]pyrimidine) and THF as the solvent at 60°C yielded poly(sulfonamide urethane)s (**38**) in good-to-excellent yields, as shown in Supplementary Scheme S10 (Chen et al., 2022).

### 2.4 Solid-phase synthesis

Solution-phase synthesis (SPS) has historically been the dominant method as a foundation for the synthesis and discovery of heterocyclic bioactive scaffolds. The emergence of SPS paved the way for the development of bioactive heterocyclic compounds and distinct structural diversity. MCRs contributed toward the process of creating solid-phase protocols that could speed up the discovery of new drugs and active heterocyclic compounds. As an ongoing research field, there are a lot of incoming methods and reports in the literature on solid-phase synthesis *via* MCRs.

Potowski et al. (2020) achieved substituted α-aryl glycines (**39**) in a 3CR of amines, boronic acids, and glyoxalic acid at room temperature using DMF as a solvent, as shown in Supplementary Scheme S11. As a result, the Petasis 3CR procedure is very appealing for the manufacture of DNA-encoded small-molecule screening libraries. It was discovered that the copper (I)/bipyridine reagent system in dry organic solvents promotes the Petasis reaction.

The synthesis of 2-amino-4*H*-benzo[*b*]pyrans (**40**) was achieved from a 3CR of 4-chlorobenzaldehyde, malononitrile, and 5,5-dimethyl-1,3-cyclohexadione (dimedone) using mesoporous mPMF as a heterogeneous catalyst for 1 h at room temperature, as shown in Supplementary Scheme S12 (Zaharani et al., 2021). The reaction conditions also adhered to green chemistry principles after using the planetary ball milling process at room temperature with no solvents. This method also presents good outcomes such as a short reaction time, broad substrate scope, no sequential reactions, good yields, and the use of a safe mPMF catalyst that can be recovered for reuse. The outcome of this study demonstrated that porous organic polymers consisting of Lewis base sites with acceptor–donor hydrogen-bonding functional groups and high porosity are significant and accelerate the formation of MCRs in solid-phase reactions.

Bucci et al. (2020) reported the 4CR of metal-free 1,3-dipolar cycloaddition of cyclopentanone–proline enamines, pentanone, carboxylic acid, and sulfonyl azides for the synthesis of amidino depsipeptide (**41**), as shown in Supplementary Scheme S13. The on-resin multicomponent reaction proceeds by the formation of a primary cycloadduct, followed by ring opening and molecular rearrangement to allow a linear sulfonyl amidine function containing a peptide chain and a diazoalkane. The resultant diazo function reacts with the carboxylic group from the *N*-Fmoc-protected amino acids to form an amidino depsipeptide with a C4 aliphatic chain. This method provides stable peptide-bond bioisosteres.

Mendez et al. (2019) reported a solid phase on-resin multicomponent reaction of the amine, carbonyl, and isocyanide to obtain tetrazole-peptidomimetics (**42**), as shown in Supplementary Scheme S14. The on-resin reaction of paraformaldehyde consisted of imine formation and transimination of the resin-bound amino acid using the piperidinium ion, followed by the addition of the isocyanide component and TMSN_3_ and shaking for 72 h.

The solid-phase multicomponent reaction of chiral β-keto lactam with an aldehyde, an isocyanide, and a dienophile yielded 3-substituted isoindolinone (**43**) derivatives, as shown in Supplementary Scheme S15 (Massarano et al., 2020). Further optimization was done using microwave irradiation as the source of energy to achieve good-to-excellent yields.

### 2.5 Asymmetric catalysis

The synthesis of useful desired products, enantiopure natural scaffolds, and bioactive compounds via asymmetric multicomponent reactions remains one of the most reliable and applied methods of reactions. This method presents beneficial advantages such as a reduction in sequential steps during synthesis, atom economy, and recyclable catalysts. The development of new asymmetric MCRs is triggered by the existence of organocatalysis. Nonetheless, there are still a lot of accomplishments to be made, and several reactions that were reported still do not exhibit good chiral induction control. To overcome issues like poor enantioselectivities, new catalytic techniques and insights are needed for intervention.

The multicomponent reaction of an aldehyde, 1,3-dicarbonyl compound, and urea/thiourea using counteranion-directed catalysis (ACDC) and ionic liquid effect (ILE) at 30°C for 72 h gave rise to an enantioselective Biginelli reaction, as shown in Supplementary Scheme S16 (Alvim et al., 2018). The MCR gave excellent yield at the recommended temperatures.

Tang et al. (2018b) reported the synthesis of tetrasubstituted allenes (**45**) using a catalytic asymmetric 3CR of terminal alkynes and isatins in the presence of *α*-diazoesters. The axially chiral tetra-substituted allenoates containing a stereogenic core resulted from this one-pot synthesis, as shown in Supplementary Scheme S17. The reaction was promoted by the chiral guanidinium salt (10 mol%) and CuCl as the catalyst, with chloroform as an additive.

According to mechanism studies (Scheme 17B), the combination-acid systems (Brønsted acid combined with an assisted Lewis acid or Lewis acid mixed with an assisted Lewis acid) provide for an equally effective asymmetric environment and bring forth increased reactivity through associative contact. The acidity of the catalyst may be influenced by the acid counterions. In this regard, it is possible to speculate about the role of a Lewis acid (YBr_3_)- or Brønsted acid (HBr)-assisted Lewis acid system (L5-HBr/CuBr) for this AMCR, even if the primary function of the copper center is to produce a copper carbene intermediate rather than a Lewis acid.

Zhang et al. (2018) reported a highly enantioselective method for the synthesis of tetrahydroindolizines (**46**) from the reaction of alkenyloxindole with diazoacetate in the presence of pyrimidine. The 3CR was performed using an achiral iron (tetraporphyriinato) chloride with chiral *N,N′*-dioxide-scandium (III) complex catalysts to obtain tetrahydroindolizines in good-to-excellent yields, as shown in Supplementary Scheme S18. The reactions were carried out at mild temperatures of 30°C, with dichloromethane or methyl acetate serving as solvents.

The 3CR of indoles with 2,3-dihydropyran and methylene malonates yielded optically active tetracyclic indolines (**47a–b**) bearing four continuous stereocenters (Supplementary Scheme S19). The reactions were carried out using a copper (II) catalyst and bis-oxazoline (BOX) at 50°C with toluene and fluorobenzene as solvents. Due to the efficiency of the one-pot asymmetric multicomponent reaction, a variety of tetracyclic indoline derivatives with superior diastereo- and enantioselectivities were produced in high yields, as reported by Kuang et al. (2018).

Ji et al. (2019) reported the asymmetric catalytic reaction of benzaldehyde with α-arylidene pyrazolinones and malononitrile at −10°C using dichloromethane as a solvent and chiral cinchonine as the catalyst to produce spiropyrazolones (**48a–b**) (Supplementary Scheme S20). This method produced spiropyrazolones (**48a–b**) in good-to-high yields with better-refined enantioselectivities and good diastereoselectivities. When different solvents were tested, it was discovered that toluene, chloroform, and dichloromethane gave better yields and great diastereoselectivity of product formation; however, it was observed that there is little effect on the enantioselectivity. Solvents such as acetonitrile and tetrahydrofuran were found to be unsuitable for this type of reaction. The best temperature suitable for the reactions was found to be −10°C.

The 3CR of benzothiazole with an isocyanide derivative and dimethyl 2-benzylidenemalonate using Mg(OTf)_2_/L-RaPr_2_ as a catalyst with (CH_2_Cl)_2_ as a solvent at 35°C yielded hydrothiazole derivatives (**49**), as reported by Xiong et al. (2019) (Supplementary Scheme S21). The developed chiral hydrothiazole derivatives achieved high enantioselectivity and good yields of up to 98%.

The efficient enantioselective multicomponent reaction of 2-alkynylbenzaldehydes with amines and dimethylphosphonate in the presence of chiral silver spirocyclic phosphate acid (SPA) yielded chiral phosphonylated 1,2-dihydroisoquinoline cyclic derivatives (**50a–b**) (Supplementary Scheme S22) (Zou et al., 2020). The chiral phosphonylated 1,2-dihydroisoquinoline derivatives were achieved with high yields up of to 99% with great enantioselectivities up of to 94% enantiomeric excess (ee).

The catalyst-free multicomponent reaction of aliphatic diamines with diethyl chlorothiophosphate and diphenyl phosphoryl chloride yielded symmetric (**51**) and asymmetric (**52**) bisphosphoramidates and bisphosphoramidothioates (**53**), as reported by Zamudio-Medina et al. (2021) (Supplementary Scheme S24). The reactions provided a good yield of the desired products at low temperatures (0°C) in the presence of a trimethylamine base and dichloromethane as a solvent.

### 2.6 C-H functionalization

The carbon–hydrogen bond is the most prevalent in organic compounds; however, the formation of these bonds is relatively challenging. The synthesis and formation of this bond are more efficient and atom-economical. Practically, the initial replacement of hydrogen with a reactive functional group can be done using different approaches to permit the process of C-H functionalization. The C-H functionalization chemistry in MCRs covers the synthesis, preparation, and modification of useful scaffolds in organic chemistry.

Borpatra et al. (2019) reported the synthesis of pyrimido[4,5-*d*]-pyrimidines (**54**) from a 3CR of 6-aminouracils, aldehydes, and secondary amines in ethanol as a solvent at room temperature using acetic acid as a catalyst, as shown in Supplementary Scheme S25. The process begins with the initial reaction of 6-aminouracil and aldehyde, followed by the amine attack. The formation of the final product occurs via intramolecular α-C-H functionalization. It was discovered that when using 20 mol% of acetic acid in ethanol, the percentage yields were high at room temperature; however, when the reaction was heated under reflux, the percentage yields decreased. Their investigation further revealed that other solvents such as water, acetonitrile, dimethylformamide, and toluene were used to improve the percentage yield at room temperature.

The 3CR of 2-phenylpyridine, methyl acrylate, and ethyl bromodifluoroacetate using 1,4 dioxane as a solvent at 60°C under catalytic conditions of ruthenium(II) biscarboxylate with electron-deficient ligand P(4-C_6_H_4_CF_3_)_3_ gave better percentage yield of up to 65% for the alkylation of alkenes, as shown in Supplementary Scheme S26 (Wang et al., 2019).

A 3CR of *N-*phenyl pyrazole, crotonaldehyde, and phenyl vinyl ketone yielded (2R, 3S, E)-2-(2-(*1H*-pyrazol-1-yl)benzyl)-3-hydroxy-1-phenylhex-4-en-1-one (**56**) and 3-(2-(*1H*-pyrazol-1-yl)phenyl)-1-phenylpropan-1-one (**57**) using a Co(III) complex containing a chiral cyclopentadienyl ligand, as shown in Supplementary Scheme S27 (Herraiz and Cramer, 2021). The diastereoselective and enantioselective C-H functionalization reactions gave good percentage yields under Co(III) as a catalyst; however, when rhodium was used, the reaction never materialized. Additionally, when other solvents such as dioxane or Me-THF were used, the reactions were not efficient.

Shi et al. (2020) reported the multicomponent reaction of primary and secondary C(sp3)-H of *N*-fluoro-substituted amides in the presence of elemental sulfur and P(O)H compounds to obtain phosphorothiolation products (**58 and 59**), as shown in Supplementary Schemes S28, 29. The investigation was further done to check the effect of substituents by using substrates with electron-donating and electron-withdrawing groups on the aromatic ring, which resulted in a good improvement in the percentage yield. In addition, the scope was increased by exploring P(O)-H compounds such as HP(O)(OnBu)_2_ and HP(O)(OiPr)_2_, resulting in good yields.

The highly diastereoselective C-H functionalization multicomponent reaction of pyrrolidine, 4-hydroxycoumarin, and benzaldehyde yielded 4-hydroxy-6-methyl-2-pyroneo[2,3*-b*]pyrrole derivatives (**60**), as shown in Supplementary Scheme S30 (Mandal et al., 2018). When the reaction was performed at 120°C using toluene as a solvent, the percentage yield conversion was better than that at high temperatures of 140°C–150°C. In addition, it was discovered that using low-boiling point solvents does not improve yields. In order to improve the yields, Mandal et al. used Brønsted acids as additives.

In the presence of an acid, an *N*-heterocycle condenses with an aldehyde to produce an iminium ion, which isomerizes to a corresponding regioisomeric iminium ion and ultimately forms an enamine (A). Next, the enamine reacts with an additional aldehyde to produce an unsaturated iminium ion (B). The corresponding enamine was produced *via* the conjugate nucleophilic addition of dinucleophile to the iminium ion (C). Subsequently, the *β*-position of the amine underwent relative stereochemistry setting through intramolecular diastereoselective protonation (D). Ultimately, the thermodynamically more stable compound with the observed stereoselectivity was obtained through the diastereoselective cyclization of iminium ion (E).

Luan et al. (2022) reported the C-H functionalization multicomponent reaction of phenol derivatives in the presence of olefin and alkyl bromides using ruthenium as a catalyst with sodium carbonate, sodium acetate, and P(4-ClC_6_H_4_)_3_ under argon conditions at 120°C for 12 h to obtain functionalized phenols (**61**), as shown in Supplementary Scheme S31. The substrate scope was successfully tested for all three components, including phenols, olefins, and alkyl bromides. The scope expansion on the phenol component revealed that the aromatic substrates, including electron-donating and electron-withdrawing groups, resulted in good reactivity with a percentage yield range of 15%–71%. When the olefin substrate scope was investigated, substantially more products were obtained than in the phenol substrate scope in a percentage yield range of 22%–76%. The third component, alkyl bromide substrate, as anticipated, presented good reactivity and only produced eight examples of desired compounds with a percentage yield of 50%–81%, except for the *tert*-butyl bromide group, which was difficult to obtain.

Pillitteri et al. (2021) reported the use of polyoxometalate tetrabutylammonium decatungstate (TBADT) with sodium hydrogen sulfate in a C-H functionalization multicomponent reaction of anilines with aldehydes and a radical source in acetonitrile for 24 h, as shown in Supplementary Scheme S32. In order to investigate different conditions, acidic additives were tested and found to improve the percent yield, whereas other additives, such as molecular sieves, could not improve reactivity. The scope of the components was successfully explored for the radical source, giving 12 examples of desired products (**62**) with a percentage yield ranging from 20% to 82%, whereas aldehydes were reported to give 12 examples with a percent yield in the range of 20%–85%. The anilines were reported to give the highest yield of 97%, with only five examples of desired products.

The C-H functionalization multicomponent reaction of tetrahydroisoquinolines in the presence of aldehydes and aminopyrazoles/indoles using L-proline as a promoter was reported by Rahman et al. (2020), as shown in Supplementary Scheme S33. As reported, when benzoic acid, acetic acids, or Lewis acids were used as promoters, the reactivity was poor and resulted in lower percentage yields of the desired products (**63**). When L-proline was used as a catalyst at 120°C under neat conditions, even better results were achieved. The solvent effects were also tested using dimethylformamide, acetonitrile, toluene, ethanol, and dimethyl sulfoxide; however, the outcome showed that a solvent-free condition was the best for obtaining the products. It was also discovered that when the temperature was optimized, there was no improvement in yields, whereas decreasing time resulted in yield decreases. The substrate scope was investigated using various aldehydes containing electron-withdrawing and electron-donating groups.

The C-H activation multicomponent reaction of acrylic acids, formaldehyde, and malonates using rhodium as a catalyst and dichloromethane as a solvent at 130°C for 3 h was reported by Yu et al. (2021), as shown in Supplementary Scheme S34. It was reported that the reaction was triggered by the C-H activation of carboxyl-directed rhodium-catalyzed vinylic, followed by formaldehyde and malonate to yield the desired cyclopentenone products (**64**)..

### 2.7 Peptide and pseudo-peptide synthesis

Peptides are often classified as amino acid chains with up to 50 residues that are flexible (have a minimal secondary structure). Peptides are the building blocks of proteins and contain fewer amino acids than a protein. Peptides and proteins are known to be the most flexible and programmable polymers. They provide the solution to the basic biological operating principles and, in theory, permit the design of materials and structures with functions that are comparable to or even superior to those of the living world. There have been several developments in the synthesis of peptides via multicomponent reactions.

Taherinia et al. (2019) reported the multicomponent reaction of α-naphthol with malononitrile and benzaldehyde in the presence of ammonium acetate using CuNP-PNF as a catalyst at 130°C to obtain chromeno [2,3-*d*] pyrimidin-8-amine (**65**), as shown in Supplementary Scheme S35.

Moreover, the same author mentioned above further reported the multicomponent reaction of 2-bromobenzaldehydes, *p*-toluidine, and NaN_3_ in the presence of CuNP-PNF as a catalyst using solvents such as water, toluene, DMSO, or polyethylene glycol (PEG) to obtain 2*H*-indazole (**66**) at 130°C (Supplementary Scheme S36).

Martzel et al. (2020) reported the multicomponent Knoevenagel-aza-Michael cyclocondensation reaction of Meldrum with aldehydes and acids of hydroxamic acid derived from naturally occurring α-amino acids to obtain isoxazolidin-5-ones (**67**) possessing an *N*-protected α-amino acid, as shown in Supplementary Scheme S37. The reaction produced the isoxazolidin-5-ones (**67**) with good-to-high diastereoselectivities using a quinidine-derived (DHQD)_2_PHAL catalyst in dichloromethane and toluene as solvents at 30°C for 5 h. Prolonging the reactions up to 24 h allows a scope increment and overcomes the limitation of these reactions by producing up to a 99% yield of isoxazolidinone diastereoisomers, which were even possible to separate.

Nourisefat et al. (2019) described the reaction of l-histidine, 4-cyanobenzaldehyde, and barbituric acid in the presence of triethylamine (Et_3_N) as a catalyst using ethanol as a solvent under reflux conditions to produce structurally complex molecules (**68–69**), as shown in Supplementary Scheme S38. This Hantzsch multicomponent reaction consists of amino acids and peptides, of which the scope was widened by introducing other substrates such as pyrimidine, xanthene, and acridine. The reactions were investigated at various temperatures, and it was discovered that 80°C was conducive and suitable for these reactions. Different solvents such as dichloromethane, water, and toluene were tested; however, ethanol was found to be a more suitable solvent. With water as the solvent, an impressive yield was produced, although the isolated yield was said to be lower than that of ethanol.

The Ugi four-component reaction of cyclohexyl isocyanide, aniline derivatives, and 2-methoxy quinoline-3-carbaldehyde derivatives in the presence of lipoic acid yielded a series of quinoline-based peptides (**70**), as shown in Supplementary Scheme S39 (Thangaraj et al., 2018). The reactions were performed under microwave irradiation to obtain excellent yields with high-purity quinolone products using methanol as a solvent at 110°C for 15 min. Other solvents that were investigated include water, ethanol, acetonitrile, and dichloromethane, which gave relatively average yields, whereas methanol gave rise to a 90% yield of the desired product.

De la Torre et al. (2020) used an adopted method from Marigo et al. (2005) and Tiecco et al. (2007) by treating *N*-(phenylseleno)phthalimide in the presence of propanal using Jørgensen’s catalyst and *p*-nitrobenzoic acid (additive) at 1°C–20°C for 24 h in toluene to obtain benzylamine, as shown in Supplementary Scheme S40. The prepared benzylamine was used in a Ugi four-component reaction with aniline, *tert*-butyl isocyanide, and acetic acid in the presence of sodium sulfate (Na_2_SO_4_) as a drying agent to achieve selenium-based peptoid (**71**) conjugates in moderate-to-good yields.

Zarezin et al. (2018) reported the multicomponent synthesis of peptides (**72**) and depsipeptides (**73**) containing a *β*-amino acid fragment in good yields, as shown in Supplementary Schemes S42, 43. The reaction of *N*-formyl amino acid, *N*-methylmorpholine, and ethyl chloroformate in THF as a solvent at −20°C for 5 h in the presence of triethylamine yielded (*S*)-*N*-(4-diazo-3-oxobutan-2-yl)formamide (**72**) derivatives, as shown in Supplementary Scheme S41.

When an amine, isocyanide, carboxylic acid, and aldehyde were set for a reaction in dry methanol for 12 h at room temperature, (3*S*)-methyl 3-(2-(N-benzylbenzamido)-3-methylbutanamido)-4-phenylbutanoate (**73**) derivatives were obtained, as shown in Supplementary Scheme S42.

Farhid et al. (2021) reported the synthesis of pseudo-peptides (**73**) via isocyanide-based consecutive Bargellini/Ugi multicomponent reactions. The reaction of acetone, chloroform, sodium hydroxide, and isocyanides *via* the Bargellini reaction yielded 3-carboxamido-isobutyric acids, which was then followed by the Ugi multicomponent strategy using 3-carboxamido-isobutyric acids with aldehydes, isocyanides, and amides to produce pseudo-peptides (**73**) containing three amide bonds, as shown in Supplementary Scheme S43.

## 3 Conclusion

MCRs have been used for more than a century, yet it is still unclear who made the first discovery and first publication of an MCR. However, the Strecker multicomponent reaction (Strecker, 1850) (S-3CR) was described in 1850, followed by the Hantzsch (Hantzsch, 1882) dihydropyridine (DHP) synthesis reported in 1882. To date, MCRs are considered an advantageous approach because of their ability to rapidly generate molecular diversity and structural complexity of interest for different applications (Mohlala et al., 2021b; Mohlala et al., 2020).

This review demonstrates MCRs as an efficient and facile way to achieve organic compounds and new materials in applications of interest by showcasing selected approaches conveniently used in medicinal chemistry, green chemistry, and other important areas in organic synthesis during the last 5 years. The involvement of MCRs in the synthesis of bioactive scaffolds in medicinal chemistry and drug discovery paved the way for the discovery and preparation of drugs such as lidocaine (xylocaine) (Ugi and Steinbrückner, 1961), mandipropamid (Lamberth et al., 2008), nifedipine (procardia) (Bossert et al., 1981), ezetimibe (zetia) (Rosenblum et al., 1998), and prostaglandin B1 (Vazquez-Romero et al., 2009). MCRs in drug discovery continue to provide convenient ways to develop new products. Based on the IC_50_ values reported, *a*-acyloxycarboxamide compounds are safer than doxorubicin during cytotoxicity tests on normal human fibroblasts and showed anticancer activities against MCF-7 breast, NFS-60 myeloid leukemia, and HepG-2 liver cells utilizing the MTT assay. The *in vitro* antimicrobial activity evaluations of some of the compounds (pyrano[2,3-*d*]-pyrimidinone) are comparable to reference drug ciprofloxacin, and α-sulfonamido phosphonate compounds gave good MIC values of 1.56 μg/mL and 3.125 μg/mL.

The MCR approach is considered an alternative route that adheres to the green chemistry standards practiced in laboratories. MCRs offer a great chance for greener chemical production methods to realize safe environments and pleasant practices in laboratories. The use of green solvents, working at mild temperatures, utilizing biocatalysts, and engaging in catalyst-free reactions are some of the most well-known suggested practices and circumstances for a green chemical process. MCRs promote sustainable chemistry by developing synthetic methods such as electrochemical multicomponent reactions using reticulated vitreous carbon (RVC) anode/Pt plate cathode systems in acetonitrile; the use of visible light using 1,8-diazabicyclo(5.4.0)undec-7-ene (DBU) as an additive under mild conditions remains one of the safest methods discovered. Using biodegradable green solvents, such as PEG-200, and microwave-assisted reactions provides solutions adhering to green chemistry principles.

The development of polymer science and innovative materials has been greatly accelerated by MCP. These polymeric materials have distinctive mechanical, chemical, optical, thermal, fluorescence sensing, and optoelectronic features that improve their various practical chemical applications. In addition, MCRs are used in SPS and peptide synthesis for the development of bioactive heterocyclic compounds with distinct structural diversity.

On the other hand, multicomponent asymmetric reactions produce useful desired products, such as enantiopure natural scaffolds and bioactive compounds with benefits such as atom economy and recyclable catalysts, whereas C-H functionalization in MCRs can be done using different approaches. However, the formation of a C-H bond is relatively challenging in useful organic compounds. The synthesis and formation of these bonds are more efficient and atom-economical during the preparation and modification of scaffolds in organic chemistry. To date, MCRs remain a better alternative route for organic synthesis, as demonstrated in this review, and will be explored more in the future.
