# Supplementary material for: Highlighting multicomponent reactions as an efficient and facile alternative route in the chemical synthesis of organic-based molecules: a tremendous growth in the past 5 years
Source: Front Chem. 2024 Sep 18;12:1469677. doi: 10.3389/fchem.2024.1469677 (PMC11445040; doi:10.3389/fchem.2024.1469677)
Supplement: Supplementary file 1 [file DataSheet1.pdf]

Highlighting multicomponent reactions as an efficient and facile alternative route in chemical synthesis of organic based molecules: A tremendous growth in the past five years.

Reagan Lehlogonolo Mohlala [1,*], Thompho Jason Rashamuse [1] and Elena Mabel Coyanis [1]

[1]Advanced Materials Division, Mintek, Private Bag X3015, Randburg 2125, South Africa, reaganm@mintek.co.za (R.L.M.); jasonr@mintek.co.za (T.J.R.); and mabelc@mintek.co.za (E.M.C.)

* Correspondence: reaganm@mintek.co.za (R.L.M.), Tel.: +27-11 709 4824 (R.L.M.)

Supplementary information

2.1. Medicinal chemistry

2.2 Green chemistry

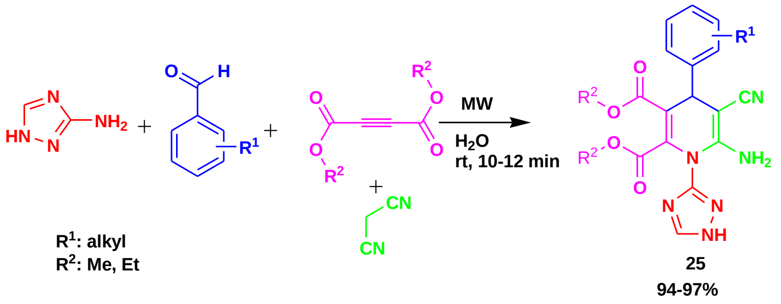

Scheme s1. 4CR for the synthesis of 1,2,4-triazole-tagged 1,4-dihydropyridine derivatives.

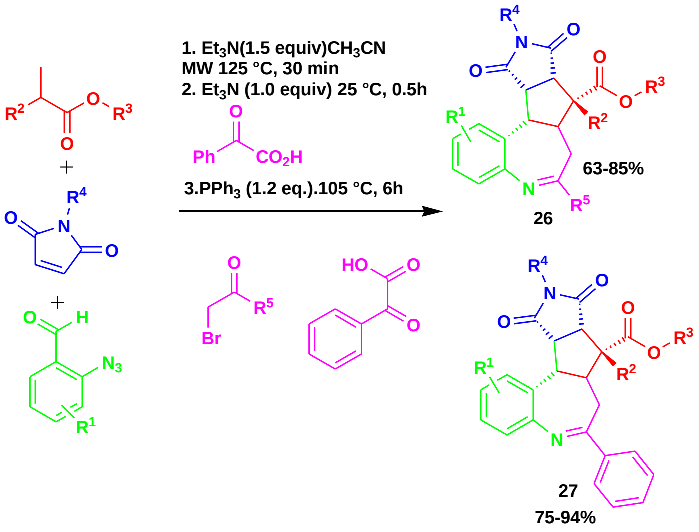

Scheme s2a. Synthesis of tetrahydro-pyrrolobenzodiazepine derivatives.

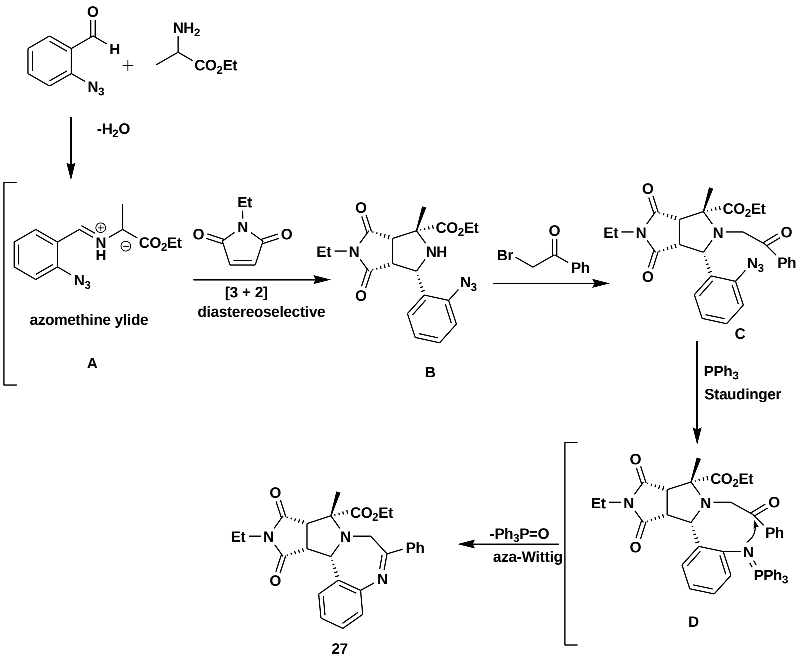

Scheme s2b. Mechanism for the synthesis of tetrahydro-pyrrolobenzodiazepine derivatives.

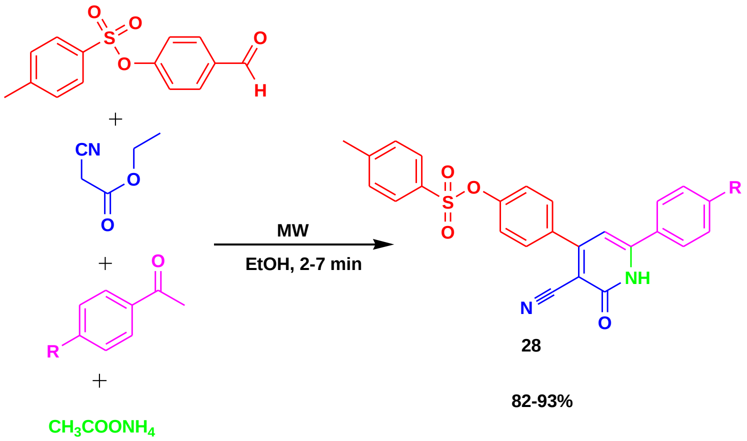

Scheme s3. Synthesis of 3-Cyanopyridines.

2.3. Polymerisations

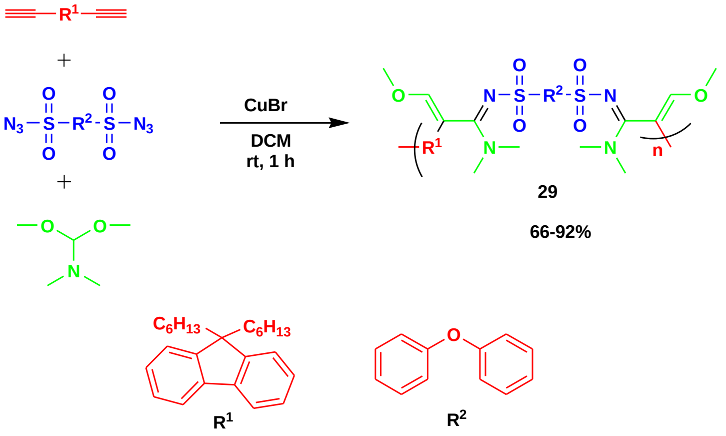

Scheme s4. Amidine-containing polymers.

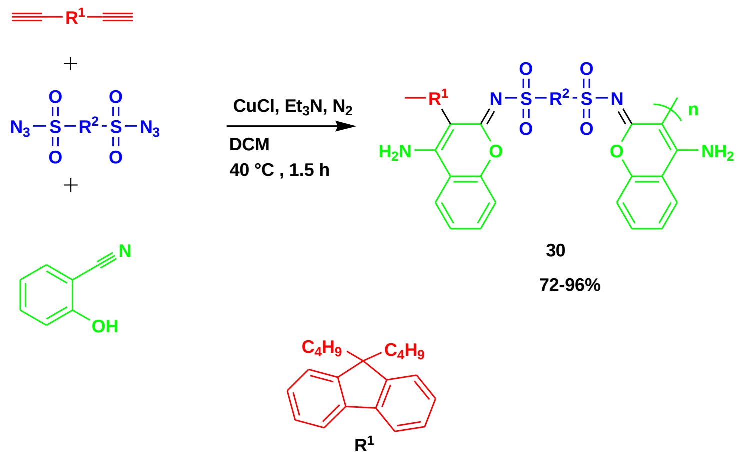

Scheme s5. Poly(*N*-sulfonylimine)s functional polymers.

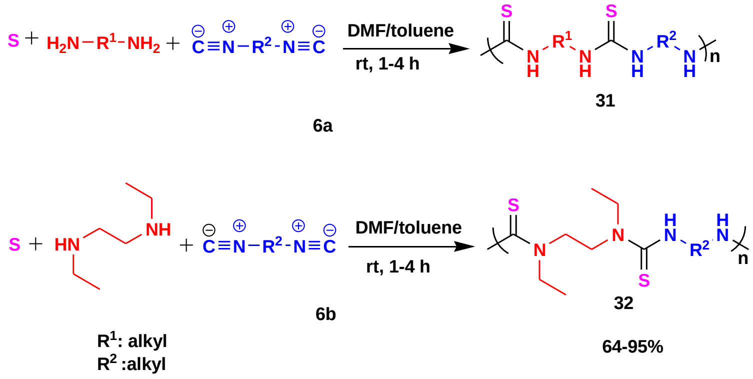

Scheme s6. a-b. Polymerisation of sulfur, diamines, and diisocyanides to form polythioureas.

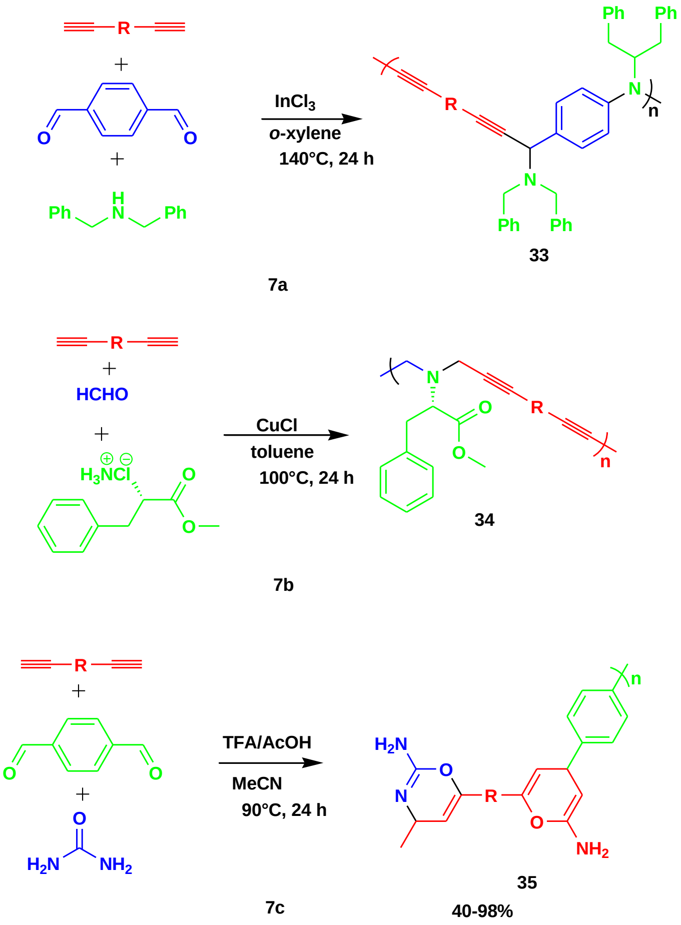

Scheme s7. a-c. Polymers with aggregation-induced emission (AIE).

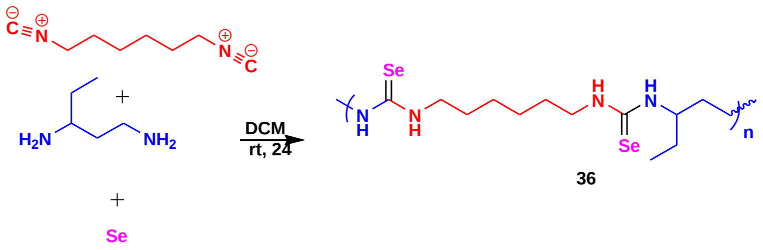

Scheme s8. 3CR of diisocyanide, diamine and selenium to form polymers.

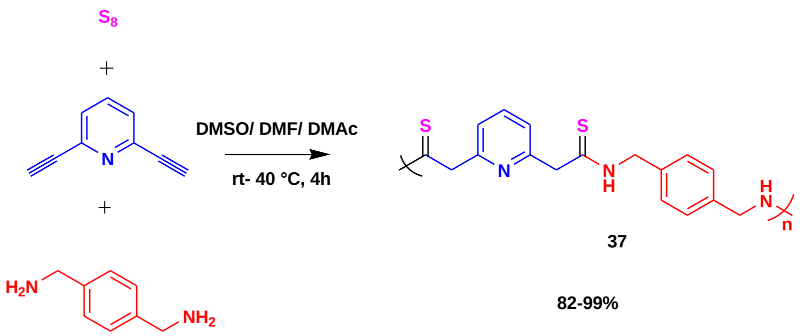

Scheme s9. Polymerisation of sulfur, alkynes and diamines to afford polythioamides.

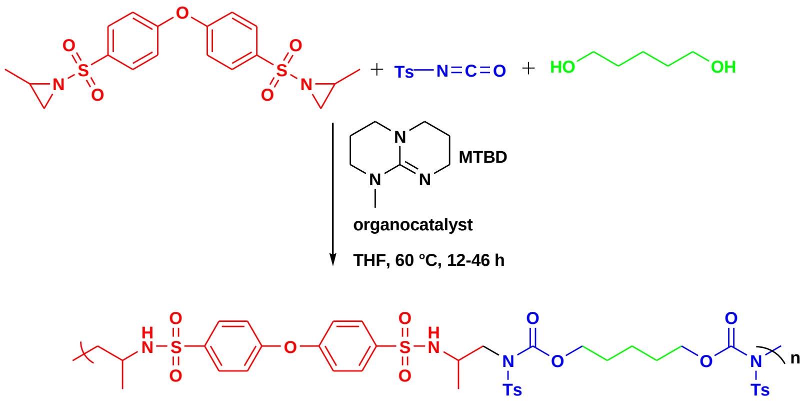

38 81-98%

Scheme s10. Synthesis of poly(sulfonamide urethane)

2.4. Solid-phase synthesis

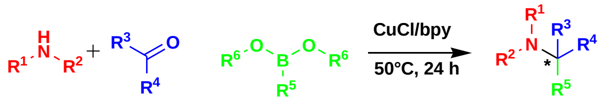

39

7-65%

R^1: DNA pyrimidines
R^2: alkyl, aryl
R^3: alkyl, aryl
R^4: alkyl, aryl
R^5: alkyl, aryl

Scheme s11. The substituted α-aryl glycines from 3CR.

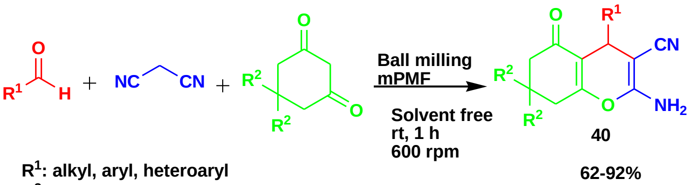

40

62-92%

R^1: alkyl, aryl, heteroaryl
R^2: H, Me

Scheme s12. Synthesis of 2-amino-4*H*-benzo[*b*]pyrans from one-pot multicomponent reaction in solid-phase reactions.

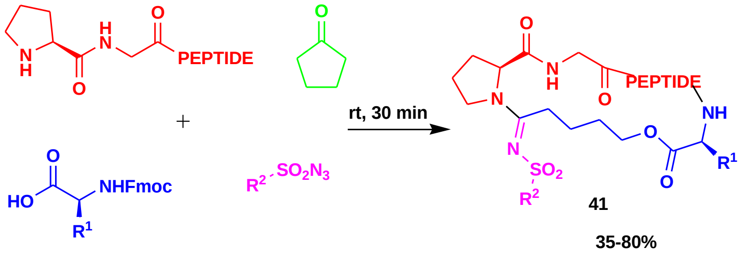

41

35-80%

R^1: alkyl, aryl
R^2: alkyl, aryl

Scheme s13. Solid phase MCR for the synthesis of depsipeptides.

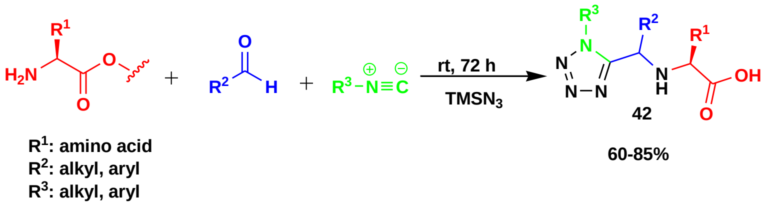

Scheme s14. Solid-phase synthesis of tetrazole-peptidomimetics by on-resin Ugi-azide-4CR.

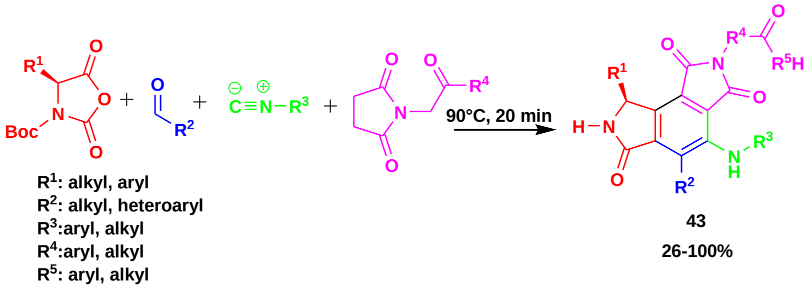

Scheme s15. Solid-phase synthesis of 3-substituted soindolinone derivatives.

2.5. Asymmetric catalysis

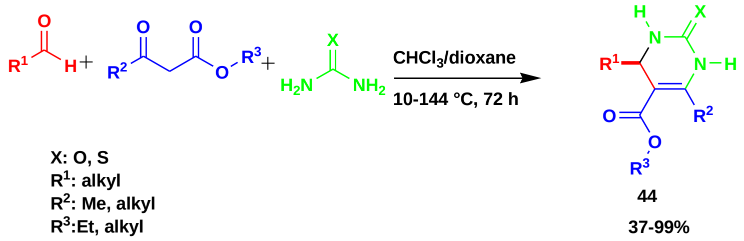

Scheme s16. Enantioselective multicomponent reaction.

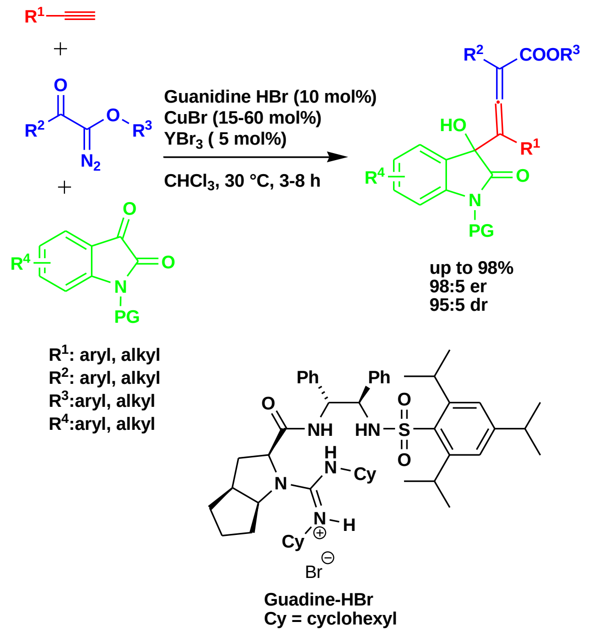

Scheme s17a. Enantioselective multicomponent reaction.

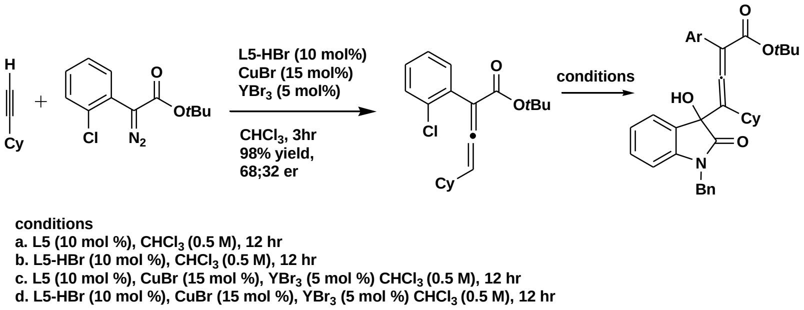

Scheme s17b. Mechanism of enantioselective multicomponent reaction.

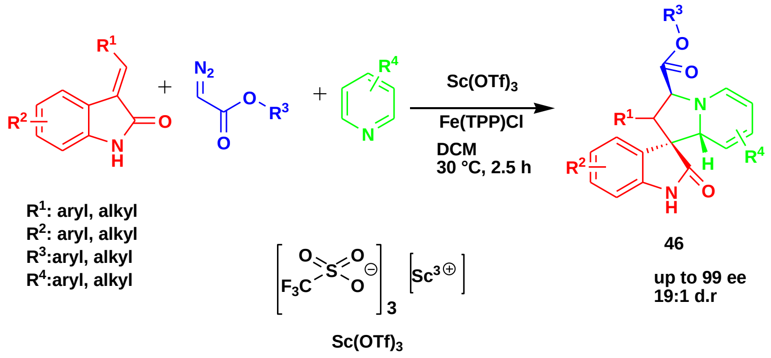

Scheme s18. High enantioselective method for the synthesis of tetrahydroindolizines.

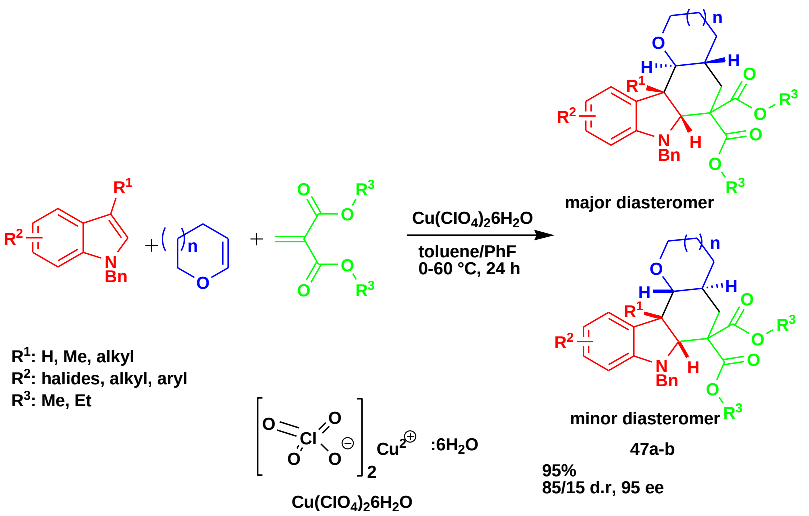

Scheme s19. The synthesis of optically active tetracyclic indolines.

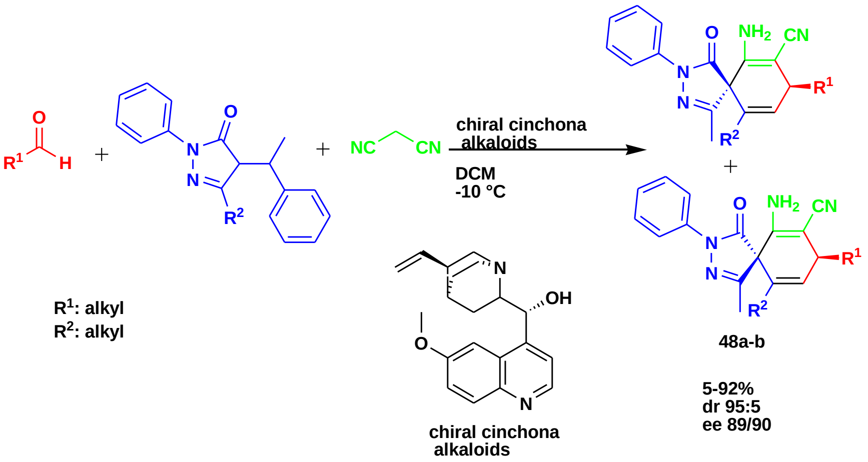

Scheme s20. The asymmetric catalytic synthesis of spiropyrazolones.

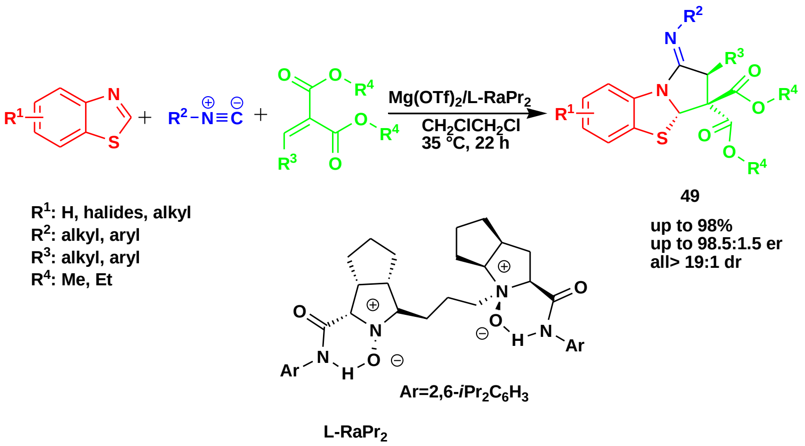

Scheme s21. The asymmetric synthesis of hydrothiazole derivatives.

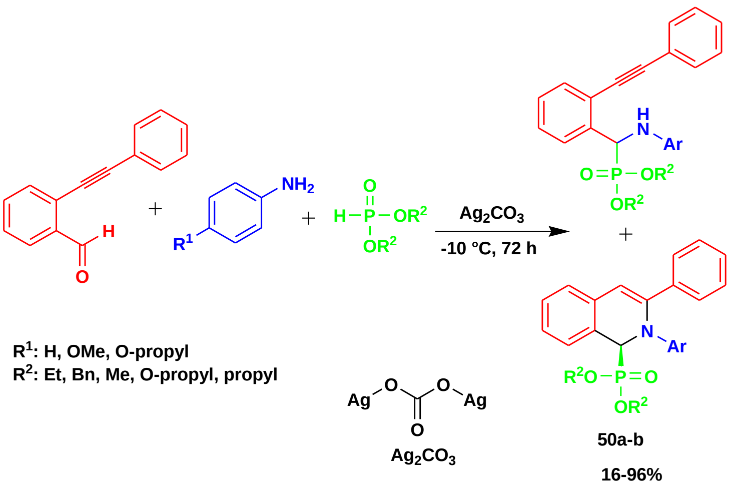

Scheme s23. The asymmetric synthesis of hydrothiazole derivatives.

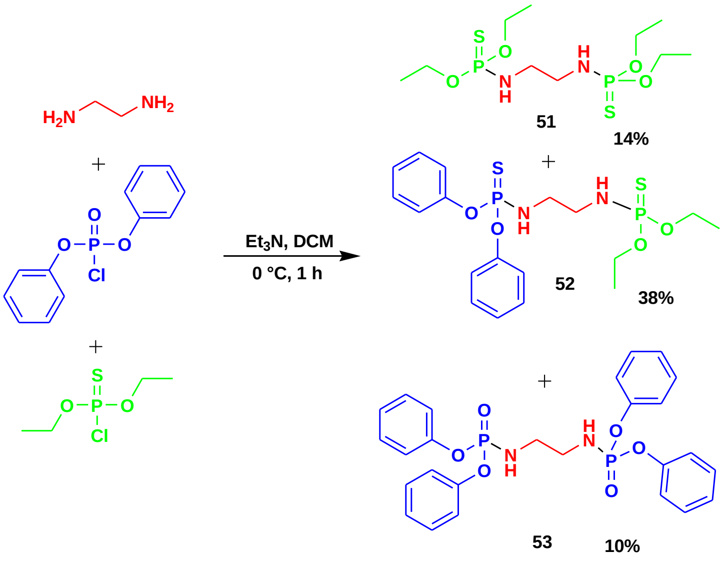

Scheme s24. The single-step synthesis of symmetric and (novel) asymmetric bisphosphoramidate and bisphosphoramidothioate derivatives.

2.6. C-H functionalisation

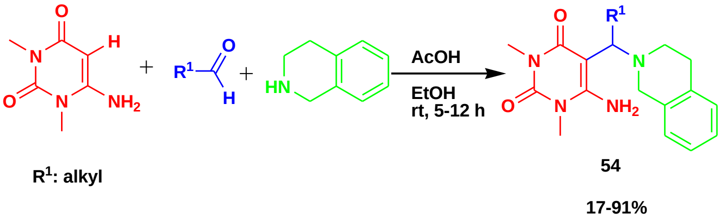

Scheme s25. The 3CR C-H functionalisation towards synthesis of pyrimido[4,5-*d*]-pyrimidines.

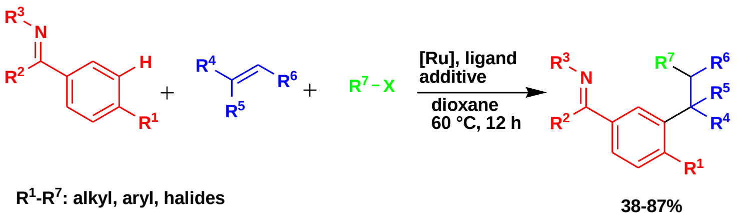

Scheme s26. The 3CR C-H functionalisation using ruthenium as a catalyst.

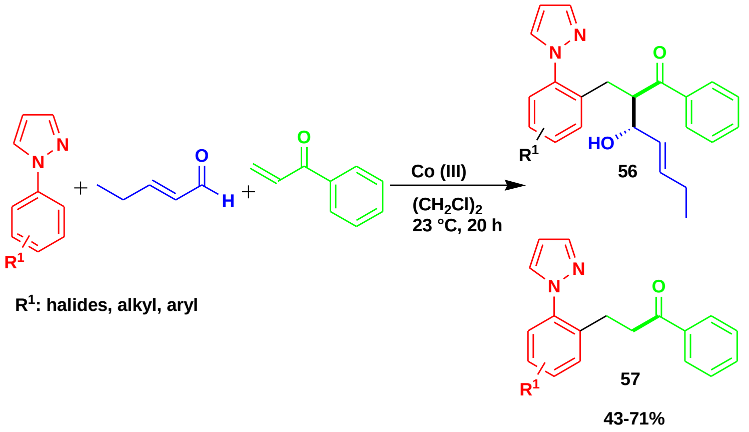

Scheme s27. The diastereoselective and enantioselective C-H functionalisation reaction.

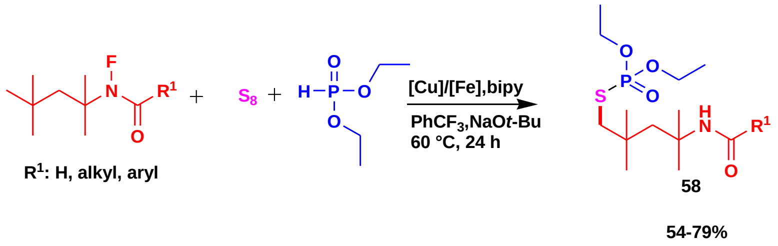

Scheme s28. Multicomponent phosphorothiolation.

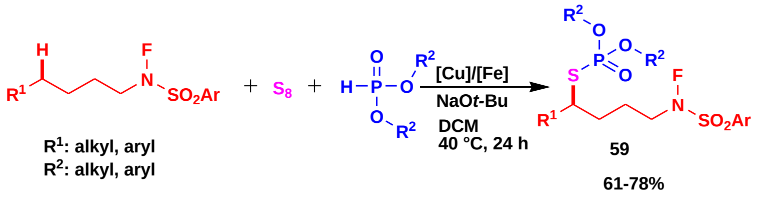

Scheme s29. Exploring the multicomponent phosphorothiolation.

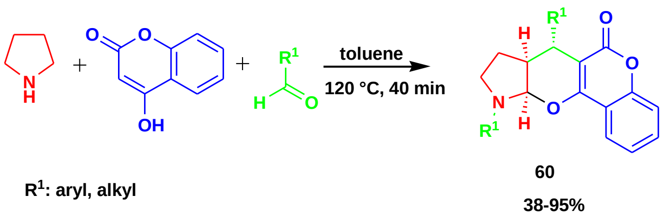

Scheme s30a. Metal-free C-H functionalisation multicomponent reaction.

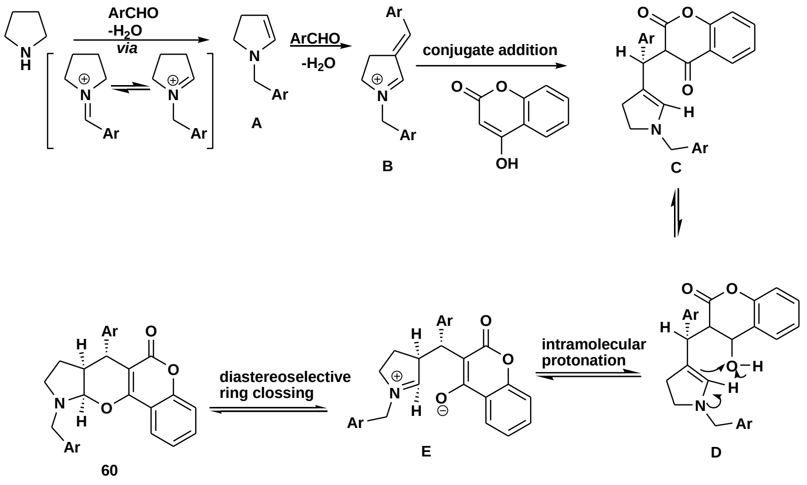

Scheme s30b. Mechanism for metal-free C-H functionalisation multicomponent reaction

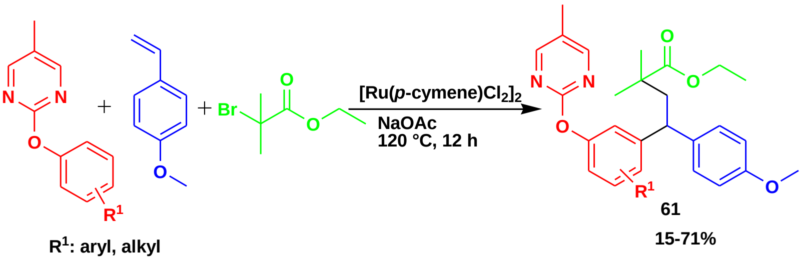

Scheme s31. The C-H functionalisation of phenol derivatives

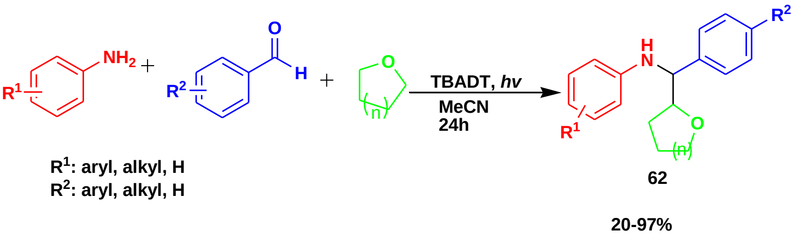

Scheme s32. The C-H functionalisation multicomponent reaction of a radical source.

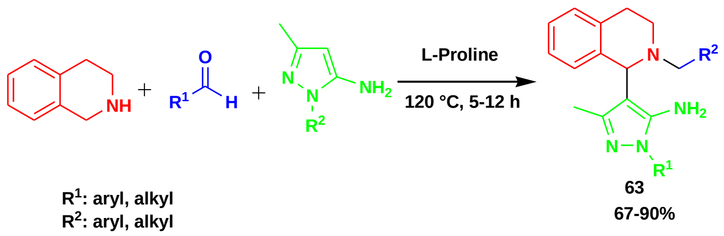

Scheme s33. The C-H functionalisation multicomponent reaction of tetrahydroisoquinolines.

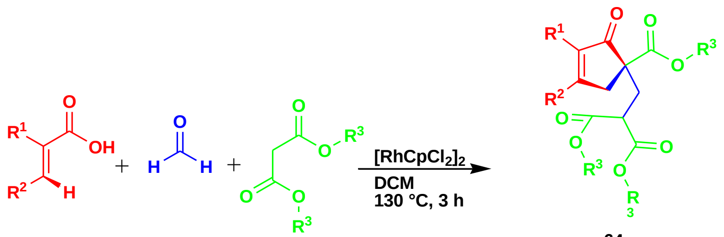

R1-R3: aryl, alkyl, H

64

42-72%

Scheme s34. Synthesis of cyclopentenones using C-H functionalisation multicomponent reaction.

2.7. Peptide and pseudo-peptide synthesis

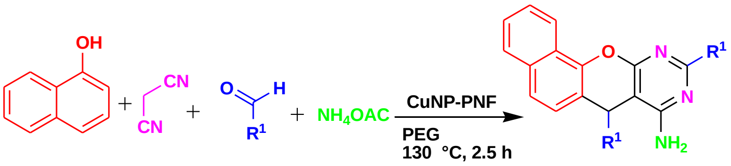

R1: aryl

65

80-92%

Scheme s35. Multicomponent synthesis of chromeno [2,3-*d*] pyrimidin-8-amine.

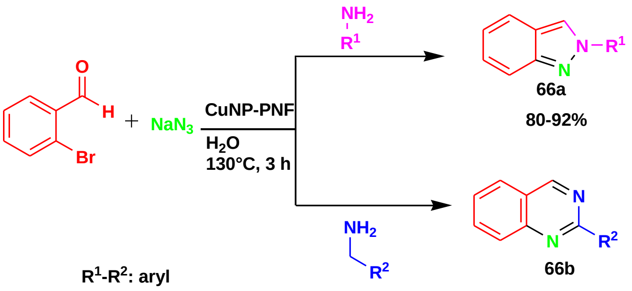

R1-R2: aryl

Scheme s36. Synthesis of indazole.

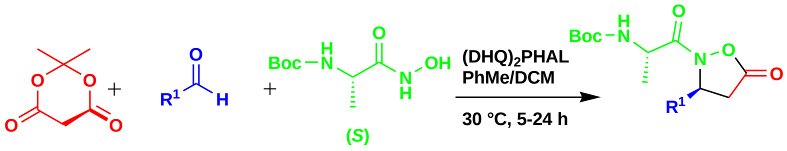

R1: aryl

(*S*,*S*)

67

20-74%

Scheme s37. Multicomponent reaction for the synthesis of isoxazolidin-5-ones.

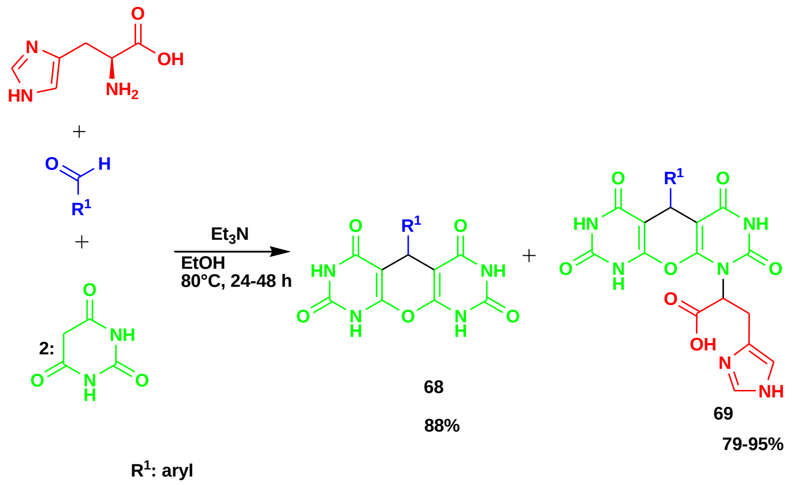

Scheme 39. Hantzsch multicomponent reaction of l-histidine, 4-cyanobenzaldehyde and barbituric acid.

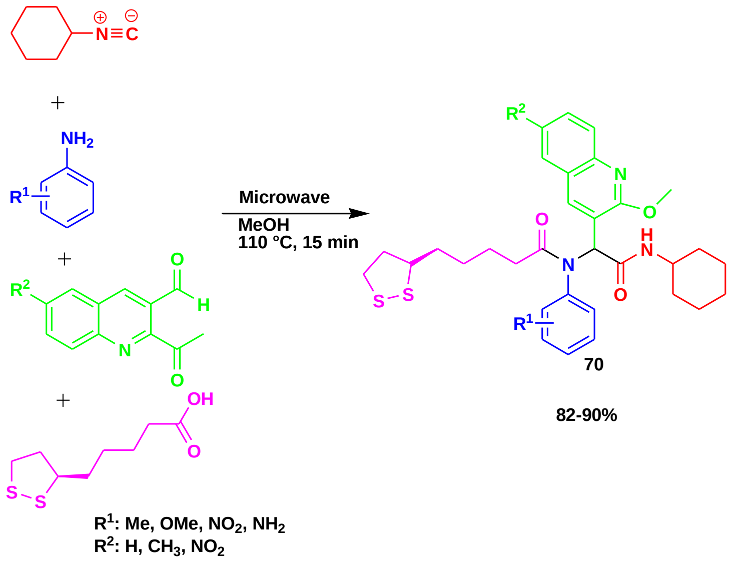

Scheme s39. The Ugi-four component reaction for the synthesis of quinolines.

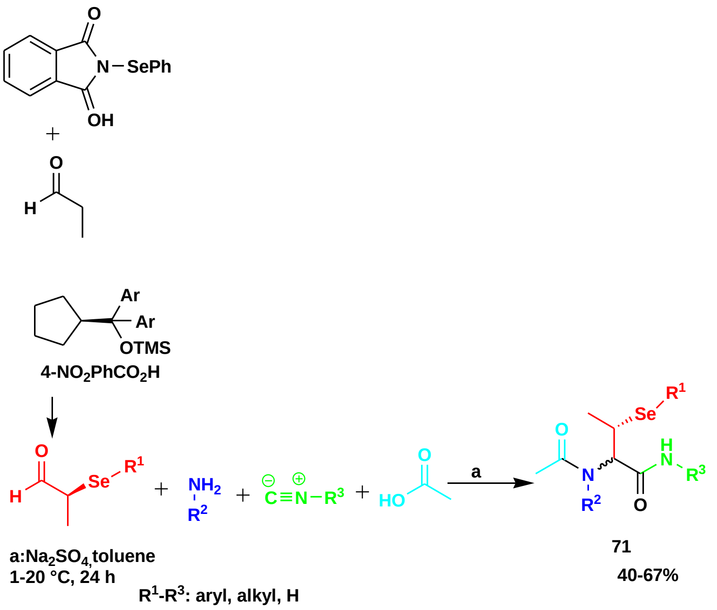

Scheme s40. Ugi-4 component reaction for the synthesis of peptoids and peptides.

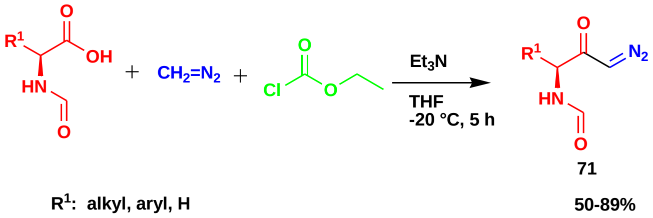

Scheme s41. Multicomponent synthesis of diazoketones.

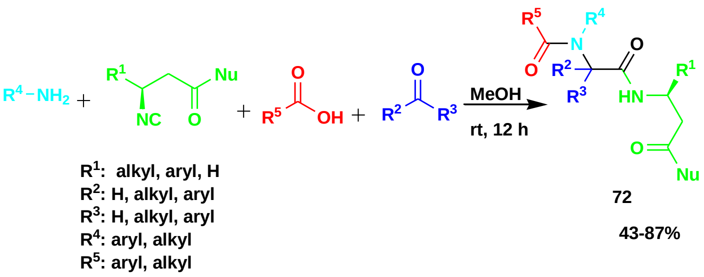

Scheme s42. Ugi-4 multicomponent for synthesis of peptides.

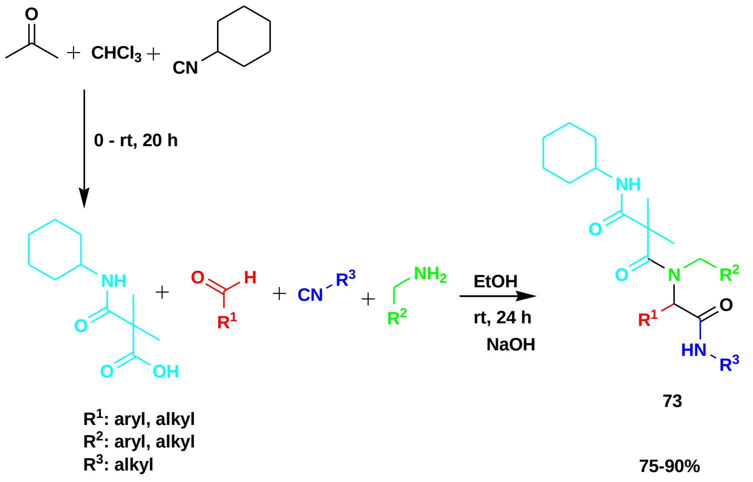

Scheme s43. Synthesis of pseudo-peptides.
